# Patient‐Derived Variants Define Constraints for Ligand Binding at the PDZ Domain of CASK

**DOI:** 10.1111/jnc.70303

**Published:** 2025-12-01

**Authors:** Debora Tibbe, Hans‐Hinrich Hönck, Neha Bhatia, Tina Truong, Lydia Proskauer, Xilma Ortiz‐Gonzalez, Jean Ann Maguire, ChangHui Pak, Hans‐Jürgen Kreienkamp

**Affiliations:** ^1^ Institute for Human Genetics University Medical Center Hamburg‐Eppendorf Hamburg Germany; ^2^ Division of Genetics and Genomics Boston Children's Hospital Boston Massachusetts USA; ^3^ Department of Biochemistry & Molecular Biology University of Massachusetts Amherst Amherst Massachusetts USA; ^4^ Division of Neurology Children's Hospital of Philadelphia Philadelphia Pennsylvania USA; ^5^ Department of Neurology and Pediatrics, Perelman School of Medicine University of Pennsylvania Philadelphia Pennsylvania USA; ^6^ Center for Cellular and Molecular Therapeutics Children's Hospital of Philadelphia Philadelphia Pennsylvania USA

**Keywords:** CASK, mutation, neurodevelopmental disorder, PDZ domain, presynapse

## Abstract

Genetic variants in the X‐chromosomal gene coding for the calcium−/calmodulin‐dependent serine protein kinase (CASK) are associated with a neurodevelopmental disorder. CASK is a member of the membrane‐associated guanylate kinase (MAGUK) family of proteins. It acts as a scaffold at presynaptic sites, as a regulator of the transport of glutamate receptors, and as a transcriptional regulator. The PDZ domain of CASK has been reported to bind to presynaptic cell adhesion molecules such as Neurexin1‐3, CNTNAP2, SynCAM and SALM1. Structural analyses of related MAGUKs indicate that the canonical SH3 and GK domains combine with the PDZ domain to form the so‐called PSG supramodule. Conserved aromatic residues (Y723 and W914) flanking the GK domain contribute to the formation of a dimeric structure of two PSG modules, which is required for high‐affinity binding to the type 2 PDZ ligand motif of, for example, Neurexin. Here we identify previously uncharacterized patient variants in the SH3 domain of CASK (I672V; P673L), which alter the intermolecular binding pocket for Y723. Both variants interfere with the binding of Neurexin‐1β, in a manner similar to the previously reported Y723C variant. Intriguingly, binding to the type 1 PDZ ligand of the cell adhesion molecule SALM1 is not altered. Using a set of highly selective patient variants, we show that the binding of SALM1 to CASK is actually not mediated by the CASK PDZ domain or the PSG supramodule, but depends on other type 1 PDZ domain‐containing proteins such as SAP97 and Veli, which associate with CASK through its L27 domains. Our data underline the relevance of an intact PSG tandem of CASK for human health.

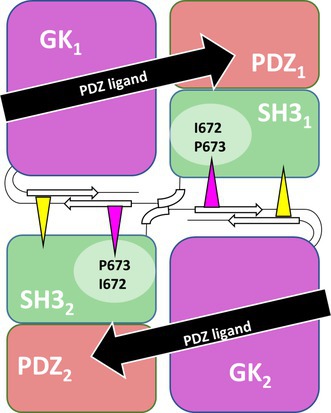

## Graphical abstract

Variants in the X‐chromosomal gene coding for the calcium−/calmodulin dependent serine protein kinase (CASK) are associated with a neurodevelopmental disorder. CASK binds through its PDZ (PSD‐95, *discs large*, ZO‐1) domain to the PDZ ligand (black arrow) of presynaptic cell adhesion molecules of the Neurexin family. We identify variants P673L and I672V in the CASK SH3 domain in patients affected by this disorder. All variants disrupt binding to Neurexin, suggesting that the SH3 domain assists in binding events at the PDZ domain. Our data support a structural model which indicates that the C‐terminal domains (PDZ, SH3, and GK) of CASK dimerize and cooperate in forming this binding site. Genetic variants in *CASK* may interfere with this assembly.

**Abbreviations**
ADHD: attention deficit hyperactivity disorder
BDNF: brain derived neurotrophic factor
CASK: calcium−/calmodulin dependent serine protein kinase
CNTNAP2: contactin associated protein 2
DMEM: Dulbecco's modified Eagle medium
ECL: enhanced chemoluminescence
GFP: green fluorescent protein
GK: guanylate kinase
GST: glutathione S‐transferase
HA: heme agglutinin
hESCs: human embryonic stem cells
IP: immunoprecipitate
iPSC: induced pluripotent stem cell
KO: knockout
MAGUK: membrane associated guanylate kinases
MICPCH: microcephaly with pontocerebellar hypoplasia
mRFP: monomeric red fluorescent protein
Nrxn1: Neurexin 1
Pals1: protein associated with LIN7 1
PDZ: PSD‐95/discs large/ZO1
PMSF: phenylmethylsulfonyl fluoride
PSG: PDZ‐SH3‐GK
RIPA: radioimmunoprecipitation assay
RRID: Research Resource Identifier (see scicrunch.org)
SALM1: synaptic adhesion‐like molecule 1
SAP‐97: synapse associated protein of 97 kDa
SH3: src homology 3
SynCAM: synaptic cell adhesion molecule
TBS‐T: Tris‐buffered saline containing 0.1% Tween
WT: wild type

## 1 Introduction

Loss‐of‐function variants in the X‐chromosomal gene *CASK*, which encodes the calcium‐/calmodulin‐dependent serine protein kinase (CASK), are associated with a neurodevelopmental disorder termed *microcephaly with pontocerebellar hypoplasia* (MICPCH) in females (Najm et al. 2008; Valayannopoulos et al. 2012; Moog et al. 2013). Similar variants in males lead to a complete lack of CASK protein due to the single X‐chromosome, and are associated with high lethality (Moog et al. 2015). Similarly, complete loss of *Cask* expression in a mouse model leads to neonatal lethality (Atasoy et al. 2007). On the other hand, missense variants in males are associated with a more variable clinical picture, ranging from moderate intellectual disability, possibly in combination with seizures and nystagmus, to the full MICPCH phenotype (Hackett et al. 2010). Pathogenic missense variants have been described in all functional domains of CASK (Hackett et al. 2010; Pan et al. 2021; Tarpey et al. 2009; LaConte et al. 2018), making it difficult to determine which domain or which functional aspect of CASK contributes to pathology in patients.

CASK functions as a scaffold protein in the anchoring of cell adhesion molecules, such as members of the Neurexin, CNTNAP, and SALM families, at synaptic sites (Hata et al. 1996; Gao et al. 2018; Brouwer et al. 2019). In addition, it plays a role in the transport of postsynaptic glutamate receptors (Jeyifous et al. 2009) and as a transcriptional regulator (Hsueh et al. 2000). CASK belongs to the superfamily of membrane‐associated guanylate kinases (MAGUKs). MAGUKs are characterized by a triad of PDZ, SH3, and guanylate kinase (GK) domains, the so‐called PSG supramodule or PSG tandem (Hata et al. 1996; McGee et al. 2001; Li et al. 2014). CASK is a unique MAGUK as it carries an N‐terminal kinase domain. Both this kinase domain, and the two L27 domains of the protein, are involved in crucial interactions with other presynaptic proteins such as Mint1, Veli family members, and Liprin‐α proteins (Butz et al. 1998; Spangler et al. 2013; Wei et al. 2011).

Interaction of CASK with the C‐termini of synaptic cell adhesion molecules was initially believed to be mediated by the PDZ domain (Hata et al. 1996; Daniels et al. 1998). More recent structural work showed that the isolated PDZ domains of CASK, PALS1, and other similar MAGUKs bind to their ligands only with low to moderate affinity (Li et al. 2014). High‐affinity binding is observed within the context of the full PSG supramodule. In this high‐affinity binding state, the PSG module exhibits a U‐shaped conformation, which enables dimerization via intermolecular interaction of two PSG units. Dimerization is driven by two conserved aromatic residues (Y723 and W914 in CASK), which insert their side chains into hydrophobic pockets of the respective other PSG module. This positions the GK domains of each PSG supramodule in proximity to the PDZ domain, and enables it to provide part of the binding interface for the PDZ ligand.

In previous work, we demonstrated the relevance of the intact CASK PSG tandem for human pathology. Analysis of patient‐derived missense variants Y723C and W914R showed that the substitution of these two large aromatic residues completely disrupts the binding of CASK to Neurexin‐1, thus confirming the relevance of the U‐shaped conformation of the PSG module for CASK function (Pan et al. 2021). So far, it remains unclear how substitutions in the SH3 domain affect Neurexin binding and CASK function in general. Here, we analyze new patient variants affecting the SH3 domain of CASK. We show that the hydrophobic pocket for Y723 in the SH3 domain is indeed essential for Neurexin binding. Furthermore, we dissect the relevance of the PSG supramodule for the interaction of CASK with different cell adhesion molecules.

## 2 Results

### 2.1 Patients

We report two patient variants in *CASK*. Patient 1 is a 17‐year‐old male affected by autism spectrum disorder, ADHD, and global developmental delay (see clinical Table for details). We identified the variant c.2014A>G; p.I672V which is maternally inherited from a seemingly unaffected mother. We also noted that two non‐related entries exist for this variant in the ClinVar database (https://www.ncbi.nlm.nih.gov/clinvar/variation/588519/).

**FIGURE 1:**
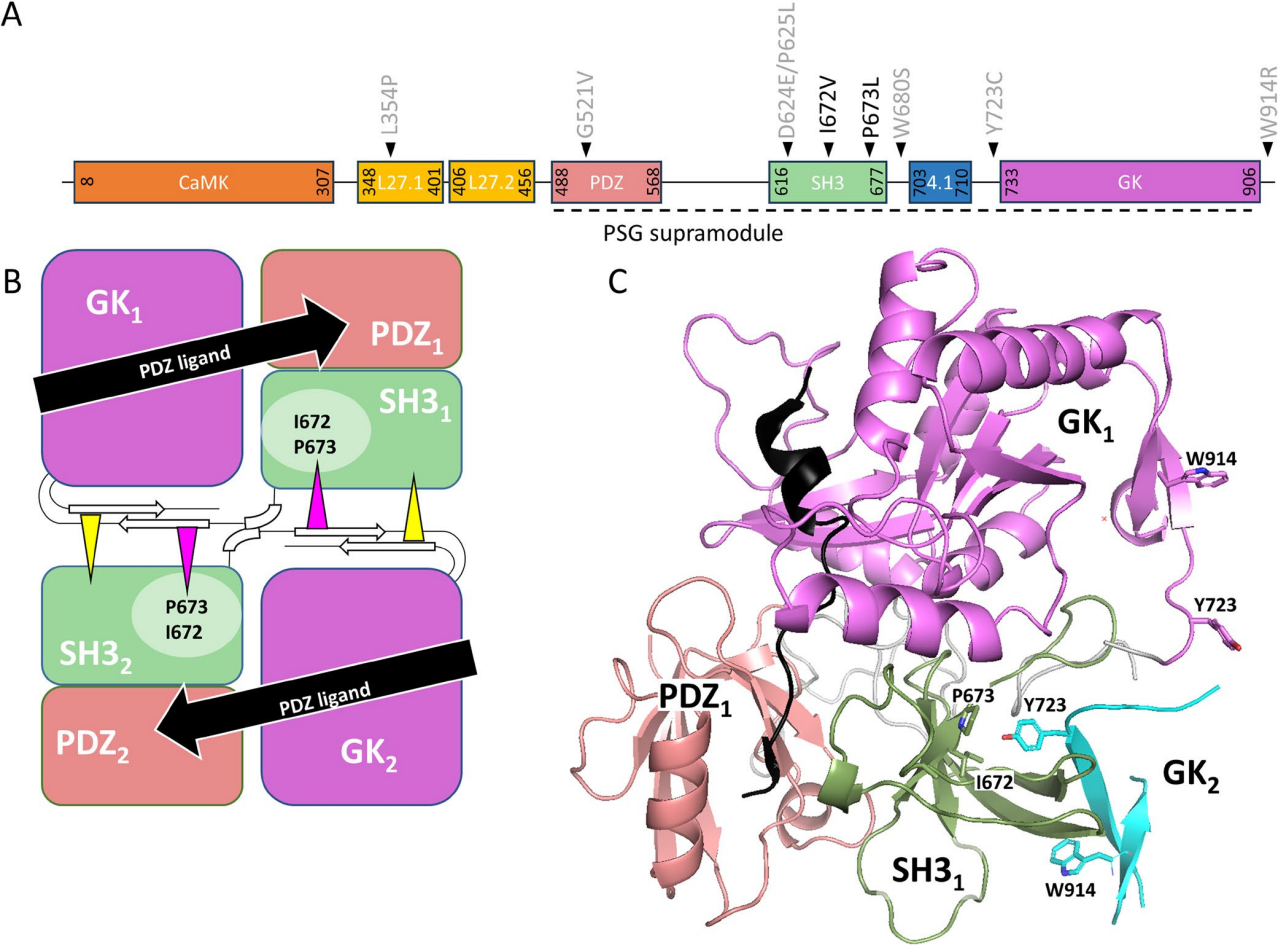
Structural models of the CASK PSG supramodule. (A) Domain structure of CASK. Patient variants identified in this study are indicated in black print, and variants previously published are shown in gray. Domains contributing to the PSG supramodule are indicated. (B) Schematic diagram of a dimer of CASK PSG tandems in the U‐shaped or PALS1‐like conformation. Amino acids altered by patient missense variants are indicated. In each molecule of the dimer, I672 and P673 are located in the SH3 domain (green) and form a hydrophobic pocket for binding of Y723 (triangle, violet) in the GK domain of the other molecule. W914 (yellow triangle, C‐terminus to the GK domain) occupies an additional hydrophobic pocket in the SH3 domain. (C) Contribution of Y723 and W914 to dimerization. The PSG_1_ supramodule (made up of PDZ_1_, SH3_1_, and GK_1_) is shown, with the aromatic residues (Y723; W914) flanking its GK_1_ domain (violet) pointing towards the SH3 domain of the second PSG supramodule (SH3_2_, not shown for clarity). The aromatic residues, flanking GK_2_ of this second PSG tandem, bind within hydrophobic pockets of the SH3_1_ domain (green) of the PSG_1_ module. I672 and P673 in SH3_1_ contribute to the hydrophobic binding pocket for Y723. This enforces a U‐shaped conformation of the PSG modul, which allows for contact of the PDZ ligand of Neurexin (black) not only with the PDZ domain (red), but also with the GK domain. The model was created by homology modeling, using the PALS1/Crumbs complex (pdb 4wsi) as a template.

The second variant, c.2018C>T; p.P673L was found in a male patient and was reported to us with questions regarding its pathogenicity. The two amino acid residues I672 and P673, altered by the patient variants, are located immediately adjacent to each other in the CASK protein (Figure 1A,C). Unfortunately, contact with the family of patient 2 was lost during subsequent functional experiments. Therefore, no personalized health information can be reported from this patient. The same variant has also been deposited in ClinVar (https://www.ncbi.nlm.nih.gov/clinvar/variation/390566/), indicating pathogenicity as it occurs in at least two apparently unrelated patients. Both variants are exceedingly rare or absent from the normal population. One male and two female carriers of the p.I672V variant are listed in the GnomAD v.4.1.0 database of 807 162 samples, whereas the p.P673L variant is absent from GnomAD.

Furthermore, for some analyses we also included the previously published variants p.D624E/P625L and p.W680S, as they affect residues within or close to the SH3 domain (Dubbs et al. 2022). Variants p.L354P, p.G521V, p.Y723C, and p.W914R have been previously characterized in much detail (Pan et al. 2021).

### 2.2 Variants in the SH3 Domain Disrupt Interactions at the CASK PDZ Domain

I672 and P673 are located in the SH3 domain of CASK. Homology modeling of the CASK PSG supramodule, based on the 3D structure of the PALS1/Crumbs complex as a template (Li et al. 2014), indicates that both residues shape the hydrophobic pocket and in fact are in direct contact with Y723 of the second PSG tandem in a dimer (Figure 1B,C).

**FIGURE 2:**
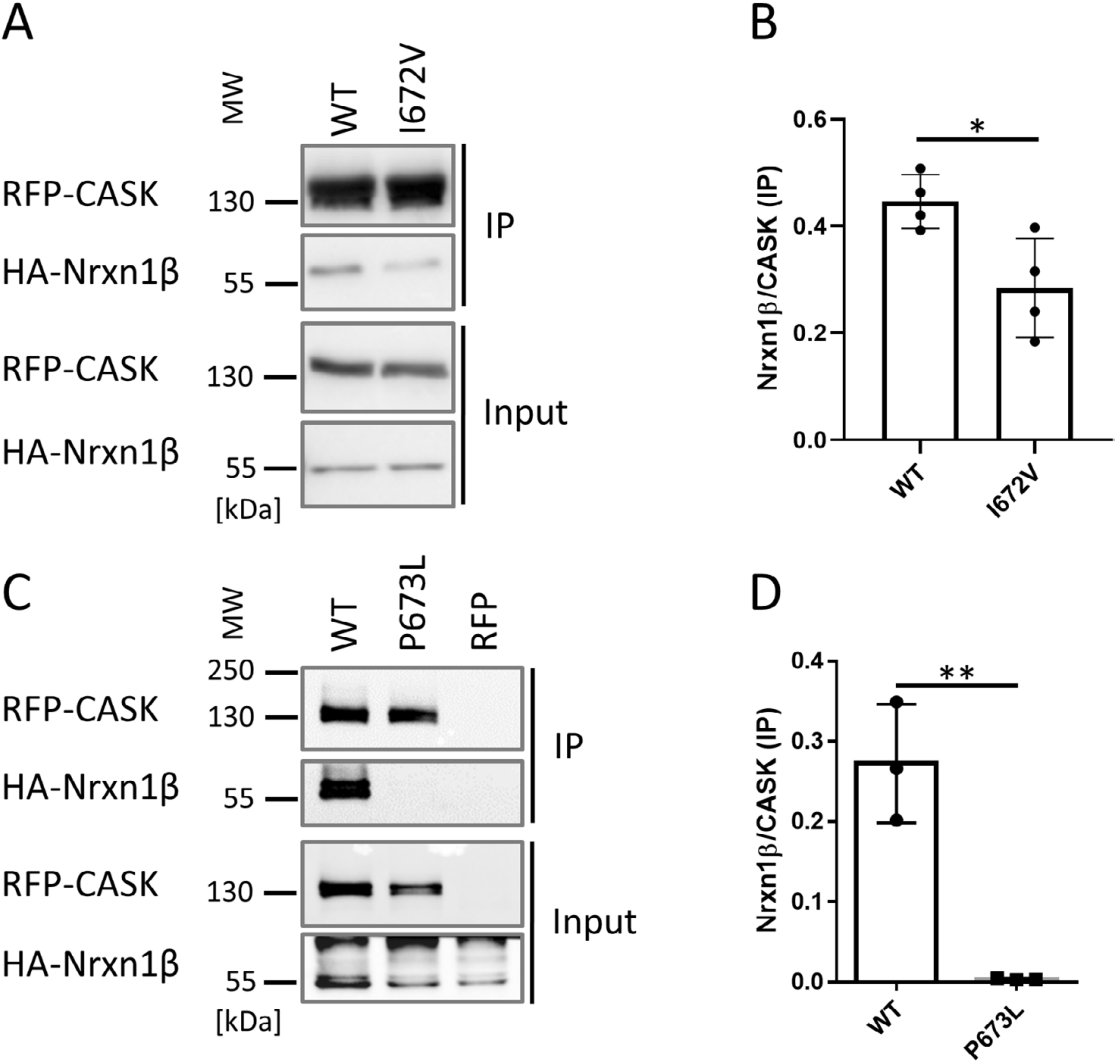
The CASK patient variants I672V and P673L are deficient for Neurexin binding. (A) HEK293T cells expressing mRFP‐CASK WT or I672V variant together with HA‐Nrxn1β were lysed 1 day after transfection. RFP‐tagged CASK was immunoprecipitated via RFP‐trap, and input and IP samples were analyzed by western blot. (B) Statistical analysis of the data shown in (A). (C) HEK293T cells expressing mRFP‐CASK WT, P673L, or mRFP alone together with HA‐Nrxn1β were lysed 1 day after transfection, and again analyzed by immunoprecipitation of RFP‐tagged proteins and Western blot. (D) Quantification of the data shown in (C). The patient variants lead to a significant reduction (I672V) or a complete loss (P673L) of Neurexin binding to CASK (Student's *t*‐test, two‐tailed and unpaired, *n* = 3 independent cell culture preparations (P673L) or *n* = 4 independent cell culture preparations (I672V), *p ≤ 0.05, **p ≤ 0.01). Error bars represent SD.

We performed coexpression/coimmunoprecipitation assays with WT and mutant, mRFP‐tagged CASK. Here we observed a partial loss of binding to Neurexin‐1β (Nrxn1β) for the I672V variant, reducing the amount of bound Nrxn1β by about 50%. For the P673L variant, we observed a complete loss (Figure 2), similar to that seen before with the Y723C and W914R variants (Pan et al. 2021). Note that Neurexin‐1β is detected in Western Blots in bands at 55 kDa, and at a higher mol. weight of about 72 kDa. This is likely due to N‐linked glycosylation, as our expression construct for this protein includes a well‐characterized glycosylation site for neurexins (Miller et al. 2011). We have quantitatively evaluated the lower of these two bands.

The difference between the two variants may be explained by the comparatively large effect that replacement of proline by a leucine may have. Due to its rigid structure, removal or introduction of a proline often has a strong effect on protein structure. In contrast, replacing isoleucine with valine is probably the most conservative exchange possible, as both residues differ by only one methylene group. This will create a slightly larger hydrophobic pocket, which is likely to produce only a moderate change in the interaction of Y723 with the hydrophobic pocket.

As these data underline the relevance of the SH3 domain for binding ligands at the PDZ domain of CASK, we included two variants here which have been recently reported in patients with a *CASK*‐related disorder (Dubbs et al. 2022). The D624E/P625L variant was observed in a boy with microcephaly and nystagmus, cerebellar hypoplasia, and seizures. The variant is inherited from his mother who carries it in a mosaic status. W680, altered in the W680S variant, is located slightly C‐terminal to the SH3 domain. It was found as a heterozygous variant in a girl with the MICPCH phenotype, suggesting that it affects CASK protein function in a rather severe way (Dubbs et al. 2022).

**FIGURE 3:**
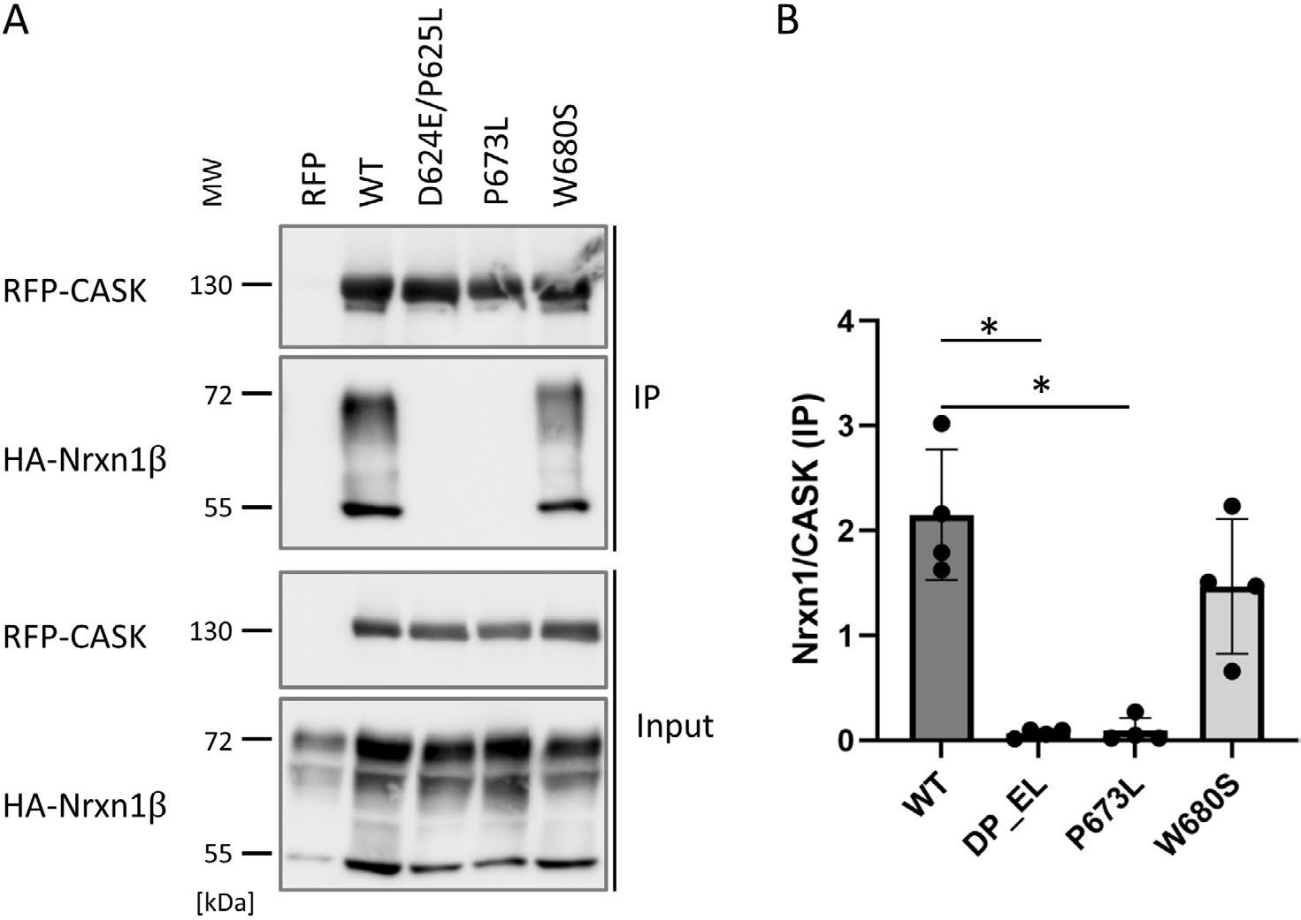
Patient variants in, or close to, the SH3 domain of CASK show distinct effects on Neurexin binding. (A) *HA* ‐Nrxn1β was coexpressed with mRFP or mRFP‐tagged CASK variants. HEK293T cells were lysed and mRFP‐containing proteins were immunoprecipitated. Input and precipitate samples were analyzed by western blotting using epitope‐specific antibodies. (B) Quantitative analysis of the data shown in A. The CASK variants D624E/P625L and P673L exhibit a complete loss of Neurexin interaction. The CASK variant W680S showed normal interaction with Neurexin, comparable to CASK WT. Kruskal–Wallis test, followed by Dunn's multiple comparison test. *p ≤ 0.05; *n* = 4 independent cell culture preparations. Error bars represent SD.

A coimmunoprecipitation experiment confirmed the relevance of the SH3 domain for Neurexin binding, as interaction was completely lost with the D624E/P625L variant. Interestingly, the W680S variant did not affect binding (Figure 3). Currently, we do not understand in which way it alters CASK function. Modeling of the CASK PSG modul carrying the variants analyzed here led to a low confidence model for this variant, particularly in the contact between the GK domain and PDZ and SH3 domains. This may suggest a possible folding defect for this variant (Figure S1).

**FIGURE 4:**
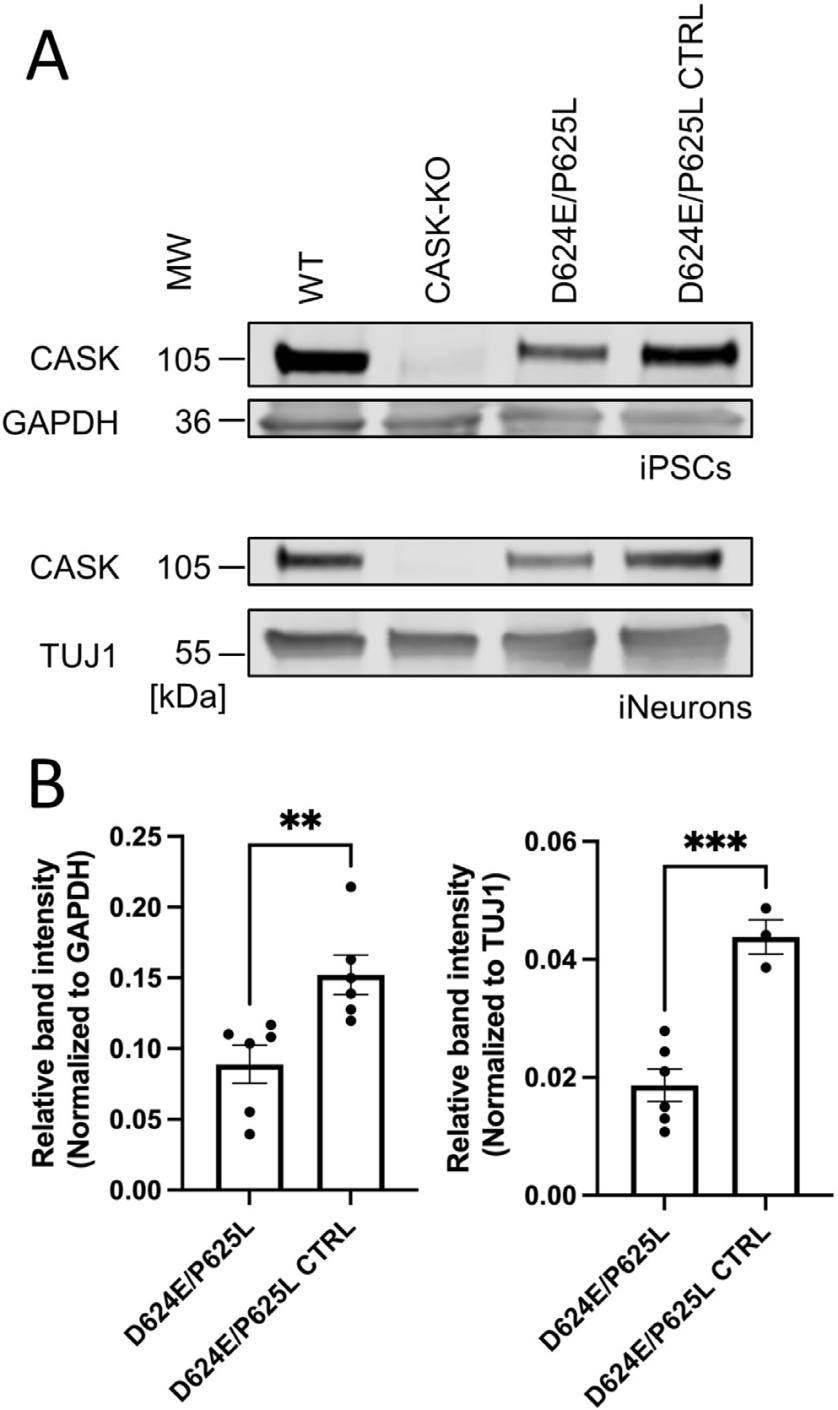
Characterization of CASK patient‐derived iPSCs and iNeurons. (A) Western Blot of lysates from iPSCs and Day 7 and Day 8 iNeurons generated from WT ESCs, CASK‐KO ESCs, (McSweeney et al. 2022), patient iPSCs carrying the D624E/P625L mutation (Dubbs et al. 2022), and a D624E/P625L CTRL line where the CASK mutation was CRISPR‐corrected in the D624E/P625L iPSCs. GAPDH was used as a loading control in iPSCs; Tuj1 was used in iNeurons. (B) Quantitation of the data shown in (A). Probing for CASK reveals diminished CASK expression in D624E/P625L iPSCs and iNeurons when compared to the corrected CTRL. Student's *t*‐test, two‐tailed and unpaired. **, ***, *p* < 0.01, 0.001, respectively. For iPSCs, *N* = 6 total replicates across three distinct culture batches for both cell lines. For iNeurons, *N* = 6 total replicates across two distinct culture batches (D624E/P625L) or three total replicates across one distinct culture batch (D624E/P625L CTRL). Error bars represent SEM.

None of the variants tested here did noticeably affect the expression levels of mRFP‐tagged CASK. However, it may be necessary to test this in a more native environment than overexpressing HEK293T cells. We had access to iPS cells from the patient carrying the D624E/P625L variant; the variant was corrected by CRISPR‐based mutagenesis, thereby generating the appropriate control iPS cell line. Additionally, we used here a human ES cell line, and a CASK KO cell line described previously (McSweeney et al. 2022). By Western blot analysis of these different cell lines, we observed that the levels of the D624E/P625L variant were reduced by about 50% when compared to the relevant control cell line, carrying the corrected CASK WT sequence (Figure 4). Thus, we conclude that variants in the SH3 domain may disrupt CASK function by preventing a conformation of the PSG tandem that is competent for binding ligands at the PDZ domain, but may also have an effect on CASK protein abundance.

**FIGURE 5:**
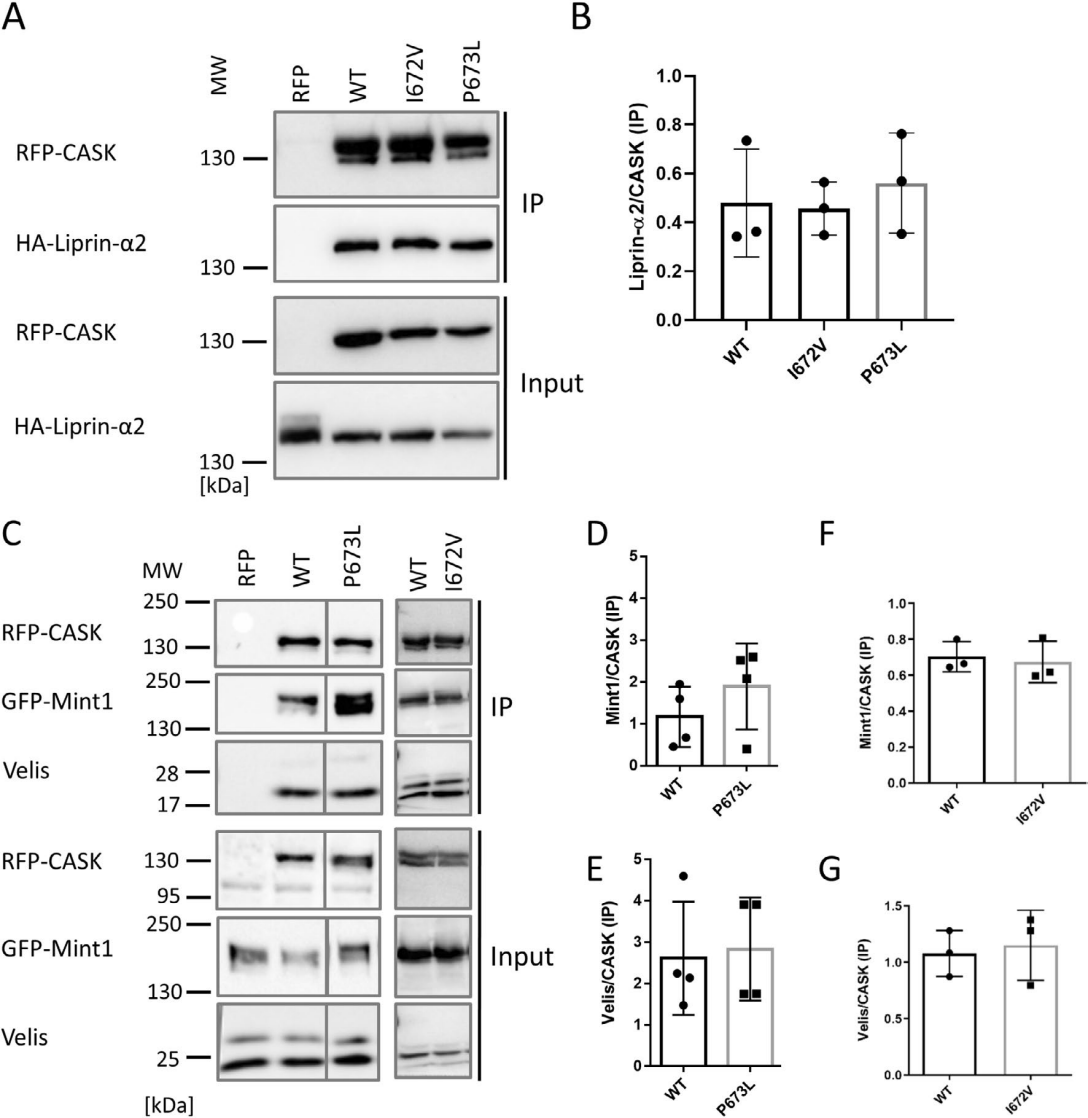
Variants in the CASK SH3 domain do not alter N‐terminal interactions of CASK. (A) mRFP‐tagged CASK variants WT, I672V, and P673L, or mRFP alone were coexpressed with HA‐tagged Liprin‐α2. RFP‐tagged proteins were immunoprecipitated from cell lysates via RFP‐trap, and inputs and precipitate samples were analyzed by Western blot using antibodies detecting the HA‐ or mRFP‐tags. (B) Quantitative analysis of four independent repeats of results shown in (A). (C) mRFP‐tagged CASK variants WT, I672V, and P673L, or mRFP alone were coexpressed with GFP‐tagged Mint1 in HEK293T cells. Proteins were immunoprecipitated from cell lysates via RFP‐trap, and inputs and precipitates were analyzed by western blot using antibodies detecting the GFP‐ or mRFP‐tags. Endogenous Veli proteins were detected using an anti‐Veli antibody. (D–G) Quantitative analysis of the results shown in (C). The P673L and I672V variants displayed no statistically significant differences in binding to Mint1 or Veli proteins compared to CASK WT; Mann–Whitney test (E) or *t*‐test (D, F, G) with *n* = 4 (WT and P673L) or *n* = 3 (I672V) independent cell culture preparations. Error bars represent SD.

In further experiments, we investigated whether the P673L variant affects other interactions of CASK. We did not see any differences when we coexpressed/coprecipitated CASK WT and the P673L mutant with Tbr1 and Cinap, nuclear partners of the GK domain (not shown). Similarly, binding to Veli proteins, interacting with the L27 domains, and Mint1, binding to the CaMK domain, were not altered for CASK P673L (Figure 5C–E). Binding to Liprin‐α2, also an interaction partner of the CaMK domain of CASK, was not altered by both CASK variants I672V and P673L (Figure 5A,B).

**FIGURE 6:**
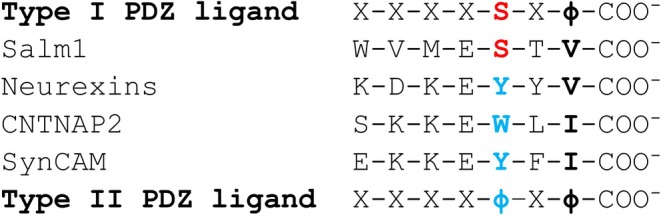
Comparison of Type I and Type II PDZ ligands of CASK interaction partners. The C‐terminal amino acid sequence of CASK interaction partners are shown, with X symbolizing a variable of residue and Φ indicating a large and hydrophobic residue. SALM1 contains a Type I PDZ ligand (red), whereas Neurexins, CNTNAP2 and SynCAMs carry Type II PDZ ligands (blue).

Having established that PDZ, SH3, and GK domains cooperate to create a high‐affinity binding site for PDZ ligands, we asked whether this type of arrangement is required for all ligands of the CASK PDZ domain. The PDZ domain of CASK is a type 2 PDZ domain, and its established ligands, such as Neurexins (Hata et al. 1996), CNTNAP2 (Gao et al. 2018), and synaptic cell adhesion molecules (SynCAMs (Biederer et al. 2002)), are known to be type 2 ligands. In addition, it was recently observed that presynaptic CASK mediates an interaction with SALM1 (Brouwer et al. 2019), which carries a type 1 PDZ ligand (Lie et al. 2018). Type 1 ligands are characterized by the amino acid sequence …X ‐ S/T ‐ X ‐ Φ ‐COO^−^, where Φ is a large and hydrophobic residue, and X is variable. Type 2 PDZ domains recognize type 2 ligands which are characterized by the C‐terminal motif, …X ‐ Φ ‐ X ‐ Φ ‐ COO^−^ (Figure 6).

**FIGURE 7:**
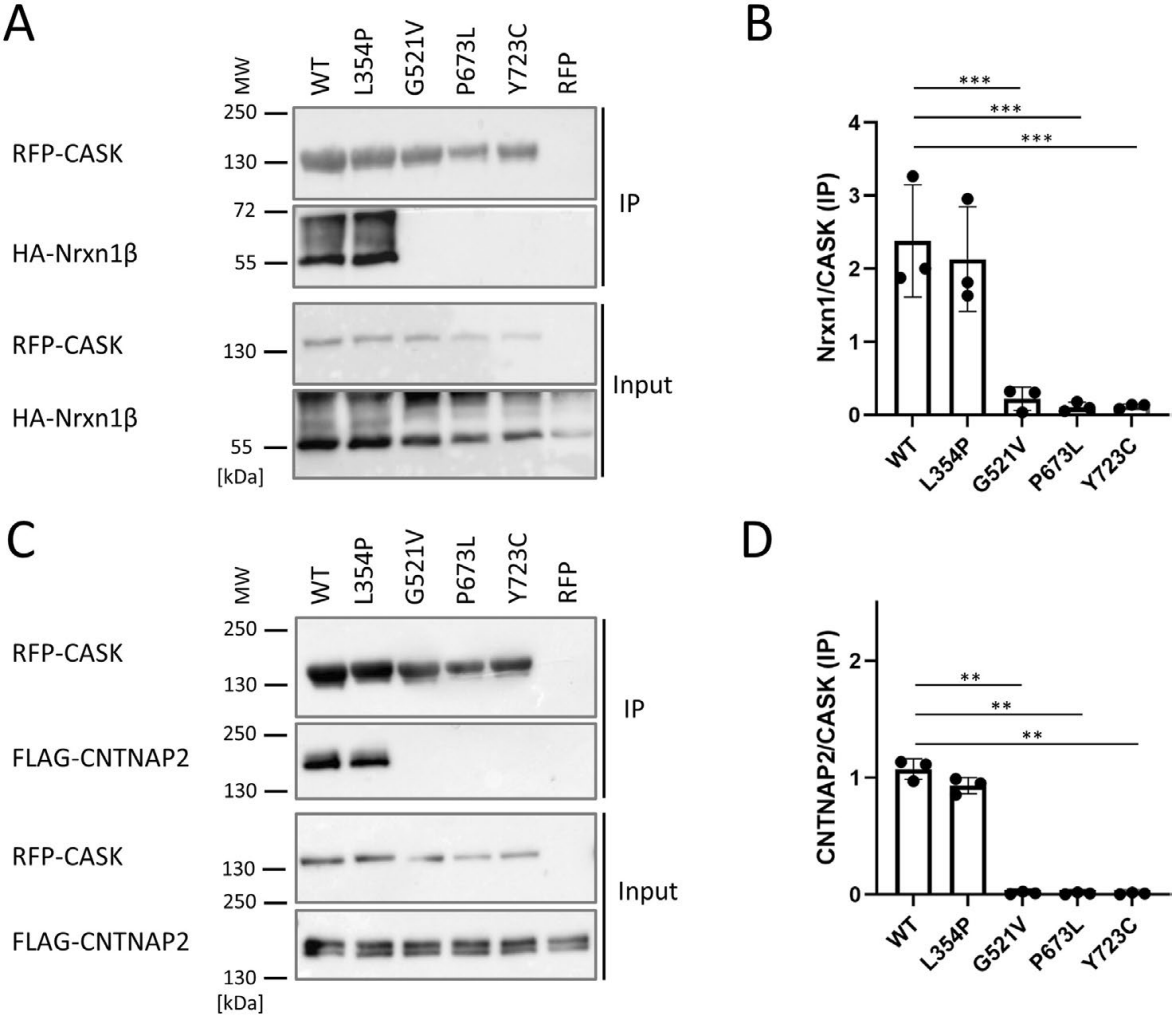
The CASK PSG supramodule variants G521V, P673L and Y723C are deficient for Neurexin and CNTNAP2 binding. (A) HEK293T cells expressing mRFP‐CASK WT, patient variants, or mRFP alone together with HA‐Nrxn1β were lysed 1 day after transfection. RFP‐tagged CASK was immunoprecipitated via RFP‐trap and analyzed by Western blot. No coprecipitation of Neurexin was detected with the G521V (PDZ domain), P673L (SH3), and Y723C (GK) mutants. CASK WT and L354P (L27.1) coprecipitated HA‐Nrxn1β efficiently. (B) Quantitative analysis of data shown in (A) (one way ANOVA with post hoc Dunnett's multiple comparisons test, *n* = 3 independent cell culture preparations, ***p < 0.001). Error bars represent SD. (C) Coexpression/coimmunoprecipitation experiments were performed with FLAG‐CNTNAP2 and analyzed again via Western blot. (D) Quantitative analysis of data shown in (B) (one‐way ANOVA with post hoc Dunnett's multiple comparisons test, *n* = 3 independent cell culture preparations, **p ≤ 0.01). Error bars represent SD.

Thus, we compared in coimmunoprecipitation experiments whether different ligands of CASK are affected by the different variants in the same manner. We used a variant in the PDZ domain (G521V, described in ref. (Pan et al. 2021)), the SH3 variant P673L identified here and the Y723C variant (Hackett et al. 2010; Pan et al. 2021). In addition, we included the L354P variant which disrupts binding of CASK to SAP97 (Hackett et al. 2010; Pan et al. 2021), a MAGUK containing several type 1 PDZ domains (Figure 1A). As expected, all mutants with substitutions within the PSG supramodule interfered with binding to Neurexin and to CNTNAP2, whereas the L354P variant, located in the first L27 domain (L27.1) of CASK, had no effect (Figure 7).

**FIGURE 8:**
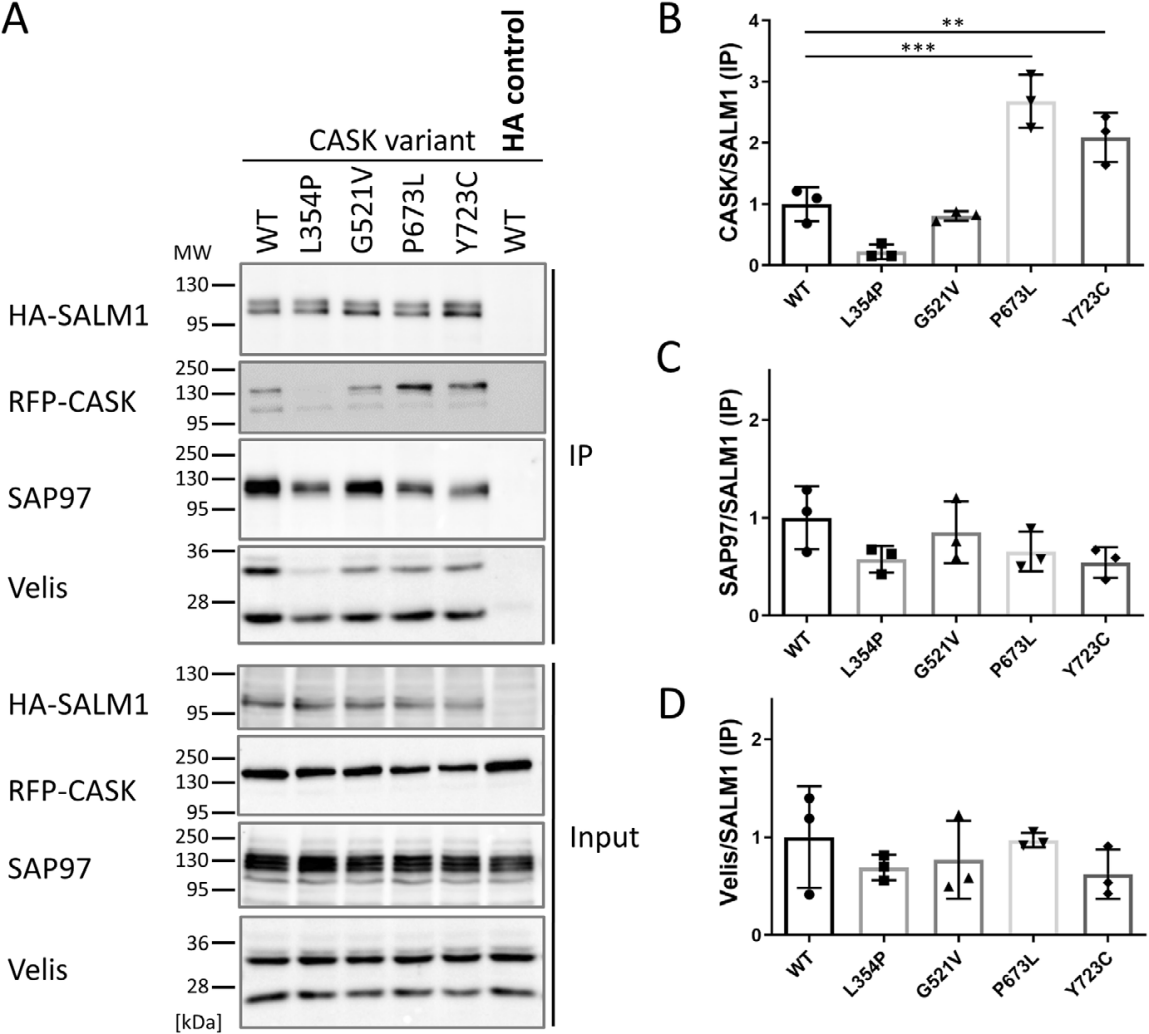
CASK variants altering the PSG supramodule interact efficiently with SALM1. (A) mRFP‐tagged CASK (WT or mutants, as indicated) was coexpressed with HA‐tagged SALM1 in HEK293T cells. After cell lysis (input samples), HA‐tagged proteins were immunoprecipitated from lysates using immobilized anti‐HA antibody (IP). Input and precipitate samples were analyzed by Western blot using the antibodies indicated. (B–D) Quantitative analysis of data shown in (A) (**p ≤ 0.01; ***p ≤ 0.001; one‐way ANOVA, followed by post hoc Dunnett's test; *n* = 3 independent cell culture preparations). Error bars represent SD.

In contrast, a strong interaction of SALM1 with CASK WT and the three variant forms (G521V, P673L, and Y723C) was observed. In fact, binding of CASK to SALM1 was increased by the P673L and Y723C variants. These data suggest that SALM1 binding does not require the U‐shaped conformation of the PSG supramodule, and possibly does not require the PDZ domain of CASK at all. As coprecipitation of CASK with SALM1 appeared to be reduced with the L354P variant, we suspected that interaction of CASK in HEK293T cells is indirect, possibly mediated by a binding partner of the L27.1 domain. Indeed, SAP97 as well as Veli family members are expressed in HEK293T cells (Tibbe et al. 2021), and are efficiently coprecipitated with HA‐tagged SALM1 (Figure 8), regardless of the CASK variant present.

**FIGURE 9:**
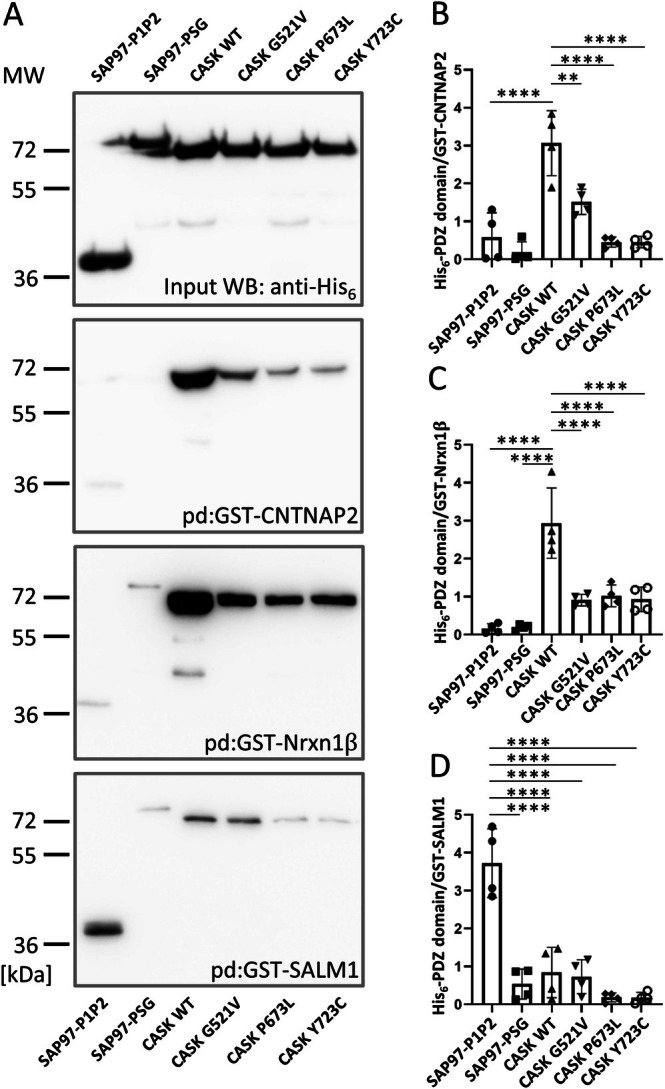
Selective interaction of PDZ ligands with MAGUK PDZ domains. (A) His_6_‐SUMO‐tagged fusion proteins of the first two PDZ domains (SAP97 P1P2) or of the C‐terminal PSG supramodule of SAP97 (SAP97 PSG), or of WT and mutant PSG modules of CASK were generated and purified from bacteria. Purified proteins were subjected to a pulldown assay (pd) with GST‐tagged C‐terminal domains of CNTNAP2, Nrxn1β, or SALM1. Input and precipitate samples were analyzed by western blotting using anti‐His_6_‐antibody. (B–D) Quantification of results. His_6_‐signals were normalized to GST‐signals derived from the same blot (one‐way ANOVA and post hoc Dunnett's test, *n* = 4, *p* ≤ 0.05 = *p ≤ 0.01 = **p ≤ 0.001 = ***p ≤ 0.0001 = ****). Error bars represent SD.

At this point, we also wished to confirm that the effects of the variants analyzed here are truly dependent on direct interactions, and not on indirect contacts mediated by interaction partners present in 293 cells, such as SAP97 or Veli proteins. For this, we prepared fusion proteins containing the PDZ ligands of Nrxn1β, CNTNAP2, and SALM1 (fused to GST), and of PSG modules and other PDZ domains in a His6‐SUMO‐tag format. In in vitro binding experiments, we observed that indeed the C‐termini of Neurexin and CNTNAP2 bind directly to the CASK PSG tandem, while the SALM1 PDZ ligand does so only weakly (Figure 9). Instead, the SALM1 C‐terminus behaves like a true type 1 PDZ ligand as it binds strongly to the fusion protein containing PDZ domains 1 and 2 of SAP97. This analysis further confirms (a) the relevance of the hydrophobic binding pocket in the SH3 domain for direct interaction of the CASK PSG module with type 2 PDZ ligands Nrxn1 and CNTNAP2; and (b) the indirect mode of interaction of SALM1 with CASK, mediated by other cellular PDZ proteins such as SAP97 (Figure 9).

## 3 Discussion

Our analysis of *CASK* missense variants presented here further confirms that loss of binding of PDZ ligands (Nrxn1β, CNTNAP2) is associated with pathology. Interestingly, partial or complete loss of binding to Neurexin or CNTNAP2, which we observe in our experimental setup, may lead to a wide spectrum of phenotypes, ranging from rather mild ID phenotype in some cases (G521V, I672V, and W914R variants, (Pan et al. 2021)), to a severe MICPCH phenotype and possibly death in other cases (M511I, M519T, and G659D variants; (Pan et al. 2021; LaConte et al. 2018)).

One consequence of the variants analyzed here may be unfolding of the PSG module. We did not observe reduced expression levels of the variants in our recombinant system. However, for one variant where patient‐derived iPSCs were available (D624E/P625L) a reduced expression by about 50% was observed at different stages of neuronal differentiation. This indicates that the patient's phenotype may be due to loss of Nrxn binding, but also to moderate loss of the CASK protein.

We have previously analyzed a variety of *CASK* variants also in neuronal cells; in particular, variants G512V, Y723C, and W914R, all of which abolished Neurexin1 binding, exhibited perfect synaptic localization similar to the WT (Pan et al. 2021). This invites the question: what is the relevance of the CASK‐Neurexin1 interaction, as it is obviously not relevant for the synaptic localization of the CASK protein? Current data from LaConte et al. (2016) suggest that CASK stabilizes neuronal levels of Neurexin1. Interestingly, inactivation of one *NRXN1* allel in human neurons leads to an increase in CASK protein levels, suggesting that both proteins are in some way dependent on each other (Pak et al. 2015). We currently assume that the CASK‐Neurexin1 interaction is crucial for a physical interaction of Neurexins with the presynaptic active zone complex, which is mediated by the binding of the CASK kinase domain to Liprin‐α proteins (Wei et al. 2011). Accordingly, we have observed that missense variants in CASK which interfere with Liprin‐α binding are associated with a particularly severe phenotype in male patients (Tibbe et al. 2022). We expect that a more thorough analysis of our iPSCs and iNeurons will help us to define which aspect of synapse formation and synaptic transmission is altered when CASK can no longer fulfill this type of bridging function.

The multitude of *CASK* missense variants found in patients allows us now to use these as tools for a better understanding of CASK interactions. We have addressed here the interactions of CASK with adhesion proteins carrying different intracellular, C‐terminal PDZ ligands. Our data indicate that Neurexins and CNTNAP2 employ the same binding site generated by the PSG tandem. Binding to both proteins is completely lost in P673L and Y723C mutant CASK, indicating a requirement for the U‐shaped conformation of the supramodule. Also, the G521V variant in the PDZ domain interferes with binding to Neurexins and CNTNAP2. On the other hand, the PDZ domain of CASK does not appear to be involved in the interaction with SALM1 at all, since all three variants that disrupt the binding of type 2 ligands (Nrxn1β, CNTNAP2) do not disrupt binding to SALM1, a type 1 PDZ ligand. In fact, P673L and Y723C increase interaction with SALM1. Our data indicate that SALM1 binding occurs indirectly through a third molecule, which is most likely SAP97. The endogenous SAP97 protein, as well as Veli proteins, are efficiently coimmunoprecipitated with HA‐tagged SALM1. CASK is coprecipitated in these experiments, and this is blocked by the L354P variant in the L27.1 domain, which we have shown previously to disrupt the CASK–SAP97 interaction (Pan et al. 2021).

Furthermore, our study shows that the full C‐terminal PSG supramodule is required for high‐affinity binding of CASK to ligands of its PDZ domain. The I672V and P673L variants described here affect residues in the SH3_1_ domain which, in a 3D structural model based on the homologous PALS1/Crumbs complex (Li et al. 2014), are in direct contact with Y723 of the GK_2_ domain in a PSG dimer. The variants would therefore be predicted to alter or destroy one of the two hydrophobic pockets in the SH3 domain. These pockets are important for maintaining the U‐shaped structure of the PSG tandem, which is required for high‐affinity binding as it enables additional contacts of the PDZ ligand with the GK domain of CASK. The U‐shaped conformation occurs upon dimerization of PSG supramodules, which hints towards a negative effect of the I672V and P673L variants on dimerization. The contribution of the SH3 domain in binding interaction partners of the PDZ domain is further underlined by the complete loss of Neurexin binding upon introduction of the D624E/P625L double mutant, as well as the G659D variant described in LaConte et al. (2018).

Finally, the increased interaction of CASK variants P673L and Y723C with SALM1, most likely caused by increased binding to SAP97, suggests that the opening of the U‐shaped conformation of the PSG tandem also alters the conformational flexibility in the N‐terminus of CASK. Both Mint1 and Liprin‐α2 bind to the kinase domain of CASK. Initial data on the Liprin/CASK interaction indicated that the L27.1 of CASK is also required for binding to Liprin‐α2 (Olsen et al. 2005), though this was later disputed (Wei et al. 2011). Work by LaConte et al. (2016) has shown that ligand binding at the PSG supramodule alters the ability of CASK to engage with N‐terminal binding partners such as Mint1 and Liprin‐α2. Thus, it appears possible that an opening of the U‐shaped conformation of the PSG motif also has repercussions on the accessibility of the L27 domains, in particular to binding of SAP97. Structural analysis of the full‐length CASK protein will be needed to clarify the molecular mechanisms behind these observations.

## 4 Material and Methods

### 4.1 Patients and Genetic Analysis

Genetic testing of patient one was performed using trio exome sequencing.

### 4.2 Expression Constructs

For expression of the CASK protein fused to an N‐terminal mRFP tag in HEK293T cells, the cDNA encoding CASK transcript variant 3 (Tibbe et al. 2021) (Addgene Cat# 23470, contributed by W. Hahn and D. Root, USA) was cloned into the pmRFP‐C1 vector using EcoRI/KpnI restriction sites. CASK patient variants were introduced using the QuikChange II site‐directed mutagenesis kit (Agilent), utilizing two complementary, mutagenic oligonucleotides. Constructs were verified by Sanger sequencing. The expression vector for HA‐tagged Neurexin‐1β (Nrxn1β) was obtained from P. Scheiffele (Addgene Cat# 58267). The construct encoding GFP‐Mint1 was provided by C. Reissner and M. Missler (Univ. of Münster, Germany). The construct for expression of FLAG‐tagged CNTNAP2 was obtained from Peter Penzes (Northwestern University, Chicago, IL) (Gao et al. 2018). The HA‐SALM1 vector (Lie et al. 2018; Li et al. 2018) was provided by Eunjoon Kim (KAIST, Daejeon, South Korea).

For the preparation of His_6_‐SUMO‐tagged CASK PSG fusion proteins, the cDNA encoding the PSG supradomain was cloned into the pET‐SUMO vector using the protocol provided by the manufacturer (Thermo Fisher Scientific). The His_6_‐SUMO vector was also used for expression of the first two PDZ domains (P1–P2) and of the C‐terminal PSG supramodule of SAP97 (SAP97 PSG). Constructs encoding GST‐tagged C‐terminal domains of CNTNAP2, Nrxn1β, or SALM1 were generated by obtaining appropriate fragments by PCR, followed by insertion into BamHI/EcoRI sites of pGEX‐4T2. Constructs were used for bacterial expression in *E. coli* BL21 cells.

### 4.3 Antibodies

1:1000 dilutions of primary antibodies were prepared in TBS‐T with 5% milk powder for Western blotting. The following antibodies were used: rb‐α‐CASK (Cell Signaling Technologies Cat# 9497S), ms‐α‐HA (Sigma‐Aldrich Cat# H9658), ms‐α‐GFP (Sigma‐Aldrich Cat# M5546), ms‐α‐FLAG (Sigma‐Aldrich Cat# F3165), rat‐α‐mRFP (ChromoTek Cat# 5f8, RRID:AB_2336064), rb‐α‐GST (Selfmade for Inst. for Human Genetics, UKE Hamburg), and rb‐α‐Veli 1/2/3 (Synaptic Systems Cat# 184002, RRID:AB_2281173). Ms‐α‐SAP97 was provided by S. Kindler (Inst. for Human Genetics, UKE Hamburg). The HRP‐coupled secondary antibodies gt‐α‐rb, gt‐α‐ms, and gt‐α‐rat (ImmunoReagents Cat# GtxRb‐003‐DHRPX, RRID:AB_2884989) were purchased from ImmunoReagents Inc. and diluted 1:2500 in TBS‐T.

### 4.4 Human iPSC Culture

H1 (WiCell) and *CASK*‐KO (McSweeney et al. 2022) ESC lines, the CASK patient iPSC line and the paired isogenic revertant control iPSC line were cultured as previously described (Pak et al. 2021, 2015; McSweeney et al. 2022). Briefly, cells were plated on Matrigel (Corning, Cat# 354234) coated plates and grown in mTesR+ media (Stem Cell Technologies, Cat# 100‐0276). Confluent cells were lifted with ReLeSR (Stem Cell Technologies, Cat# 100‐0483) and passaged every 4–5 days. Y‐27632 (10 μM; Axon Medchem, Cat# 1683) was included in the media for 24 h following passaging. All cultures were kept at 37°C with 5% CO_2_.

### 4.5 NGN2 Differentiation

iNeurons were generated from hESCs/iPSCs as previously described in Zhang et al. (2013). Briefly, confluent stem cells were lifted with Accutase (Innovative Cell Technologies, Cat# AT104‐500) and resuspended in mTesR+ (Stem Cell Technologies, 100‐0276) for counting. Approximately 3.02 million cells were transferred to a fresh tube and incubated with 20 μL of TetO‐Ngn2‐puro and rtTa lentiviruses for 5 min at room temperature. Following incubation, mTesR+ was added to the cell/lentivirus mixture to a final volume of 7 mL plus Y‐27632 (10 uM; Axon Medchem, Cat# 1683). Cells were replated on Matrigel‐coated 10 cm plates and incubated overnight. The next day, culture medium was replaced with induction media: N2/DMEM‐F12/NEAA (Thermo Fisher) containing human BDNF (10 μg/L; Stem Cell Technologies, Cat# 78005.10), human NT‐3 (10 μg/L; Stem Cell Technologies, Cat# 78074), mouse laminin (0.2 mg/L; Gibco, Cat# 23017015), and doxycycline (2 mg/L; Sigma, Cat# D9891). To select for Ngn2‐iN cells, puromycin (1 mg/L; Invivogen, Cat# NC9138068) was added to each media change the following 2 days. After 2 days of selection cells were lifted with Accutase and replated in Neurobasal medium (Gibco, Cat# 21103049) containing B27 supplement (Gibco, Cat# 17504044), Glutamax (Gibco, Cat# 35050061), human BDNF, human NT‐3, mouse laminin, doxycycline, Y‐27632, and Ara‐C (2 μM; Sigma, Cat# C6645) at a density of 7 million cells/Matrigel‐coated 10 cm plates. A full media change was performed the next day to remove Y‐27632. Two days later a half media change was performed. Cells were lifted with ReLeSR (Stem Cell Technologies, Cat# 100‐0483) and collected the following day.

### 4.6 Culture of HEK293T Cells

HEK293T cells (ATCC, Cat# CRL‐3216) were cultivated on 10 cm dishes in DMEM supplemented with penicillin/streptomycin and 10% foetal bovine serum at 37°C, 5% CO_2_ and humidified air. Cells with 70% confluence were transferred to new culture vessels. For passaging, cells were first washed with versene and detached by trypsinization. The cells were resuspended in full medium and the respective amount of cell suspension was seeded for the required confluence. This cell line is not listed as a commonly misidentified cell line by the International Cell Line Authentication Committee (ICLAC). Cell lines are authenticated by visual appearance, growth behavior and handling on passaging every week. A maximum of 20 passages is used before thawing a new batch of cells.

### 4.7 Coexpression and Coimmunoprecipitation via RFP Trap

At 40% confluence, HEK293T cells were transiently transfected using TurboFect transfection reagent (Thermo Fisher Scientific). 4 μg DNA was used for each plasmid per plate. 24 h after transfection the cells were lysed in RIPA buffer with protease inhibitors (1:500 0,125 M PMSF, 5 mg/mL leupeptin, and 1 mg/mL pepstatin A) for 15 min on ice, followed by centrifugation at 20.000 × *g* for 15 min at 4°C. 60 μL of the cell lysate was taken as input sample. The remaining cell lysate was mixed with 25 μL magnetic RFP‐Trap beads (RRID:AB_2861253), the samples were placed on a rotator for 2 h at 4°C and RFP‐tagged CASK was immunoprecipitated. Then, the beads were washed five times with RIPA buffer. The beads were resuspended in 60 μL Laemmli buffer as immunoprecipitate sample (IP). The input and precipitate samples were incubated at 95°C for 5 min and analyzed by western blotting. For quantitative analysis, the protein bands were detected by chemiluminescence using a BioRad imaging system and then quantified using ImageLab 6.0 (BioRad).

### 4.8 Immunoprecipitation of HA‐Tagged Proteins

Cotransfected HEK293T cells were lysed in IP buffer with protease inhibitors (lysis protocol similar to RFP trap). 30 μL magnetic α‐HA‐tag beads (RRID:AB_2861399) were used for immunoprecipitation of HA‐tagged proteins. After incubation with cell lysate on the rotator for 1 h at 4°C, beads were washed five times in IP buffer. Input and IP samples were analyzed using Western blotting.

### 4.9 Western Blotting

For denaturing, 5 × Laemmli buffer was added to samples to a final concentration of 1 × Laemmli. Samples were incubated at 95°C for 5 min. Equal volumes were applied to 8% polyacrylamide gels; electrophoresis was performed at 100 V initially, followed by 160 V. After separation, proteins were electrophoretically transferred to nitrocellulose membranes (Amersham Protran, obtained from Cytiva) in a wet blot apparatus (BioRad; 100 V, 90 min). Membranes were blocked in 5% milk powder in Tris‐buffered saline containing 0.1% Tween (TBS‐T) for 30 min at room temperature. Antibodies were diluted in the same solution, followed by incubation at 4°C over night on a rotator. After washing 3× in TBS‐T, membranes were incubated with HRP‐coupled secondary antibodies diluted 1:5000 in TBS‐T. After washing in TBS‐T (3 × at room temperature), detection was performed using reagents for enhanced chemoluminescence (ECL; Western Bright; Biozym, Hess. Oldendorf, Germany). Signals were detected using a BioRad imaging system, with “auto” settings, which allow for accumulation of signal until the first pixel of the image reaches saturation, thus preventing detection of oversaturated bands. Bands were quantified using Image Lab software (BioRad).

### 4.10 Bacterial Expression and Purification of His_6_‐SUMO–Tagged or GST‐Tagged Fusion Proteins

His_6_‐SUMO–tagged fusion proteins were expressed in BL21 (DE3) cells and purified from bacterial lysates prepared in native lysis buffer (50 mM NaH_2_PO_4_, 500 mM NaCl, pH 8.0) using Ni–NTA agarose beads (QIAGEN). After elution in 250 mM Imidazol, the buffer was exchanged by gel chromatography to STE buffer (100 mM NaCl, 50 mM Tris‐HCl, 1 mM EDTA, pH 8.0) using NAP25 Sephadex columns (Cytiva, Dreieich, Germany). Protein concentration was determined using Bradford reagent and BSA as a standard.

GST‐tagged fusion proteins were expressed in BL21 *E. coli* as well but lysed and purified in STE buffer, using glutathione sepharose beads following standard protocols. GST‐fusion proteins were left on the beads. For pulldown experiments, 40 μL of coated bead matrix was mixed with 20 μg of His‐SUMO fusion proteins diluted in a total volume of 1 mL IP buffer (50 mM Tris pH 8, 120 mM NaCl, 0.5% NP40, 1 mM EDTA). After incubation at 4°C for 2 h, beads were washed 3× in 1 mL IP buffer, and precipitate samples were analyzed by SDS‐PAGE/Western blot.

### 4.11 Statistical Analysis

It was performed using GraphPad Prism software (version 10.5.0, GraphPad Software Inc., Boston, MA). See Table 1 for test details. For testing significance, either *t*‐tests (comparing two experimental conditions) or ANOVA (comparing more than two experimental conditions) were used. Data were assessed for normality using the Shapiro‐Wilks test as part of the GraphPad software. Data sets failing this test were analyzed using nonparametric tests (i.e., Kruskal–Wallis test for ANOVA; Mann–Whitney test instead of *t*‐test). No tests for outliers were conducted.

**TABLE 1:** Statistics report for figures.

Figure	Type of test	Degrees of freedom	*t*‐value (*t*‐test)	*F*‐value (ANOVA)	Kruskal–Wallis statistic	Actual *p*	*p* multiple comparisons
2B	*t*‐test	6	3.036			0.0221	
2D	*t*‐test	4	6.309			0.003	
3	Kruskal–Wallis				11.87	0.0004	0.0225 (DP_EL) 0.0143 (P673L)
4B	*t*‐test	10	3.279			0.0083	
4B	*t*‐test	7	5.611			0.0008	
5B	ANOVA	8		0.2649		0.7758	0.8934 (I672V) 0.8165 (P673L)
5D	*t*‐test	6	1.163			0.2891	
5E	Mann–Whitney				U = 8	0.999	
5F	*t*‐test	4	0.3551			0.7405	
5G	*t*‐test	4	0.3334			0.7556	
7B	ANOVA	14		17.73		0.0002	0.9154 (L354P) 0.0008 (G521V) 0.0006 (P673L) 0.0006 (Y723C)
7D	ANOVA	4		341.7		< 0.0001	0.2693 (L354P) 0.0059 (G521V) 0.0058 (P673L) 0.0058 (Y723C)
8B	ANOVA	11		22		0.0003	0.8170 (G521V) 0.0006 (P673L) 0.0094 (Y723C)
8C	ANOVA	14		2		0.171	0.158 (L354P) 0.858 (G521V) 0.287 (P673L) 0.121 (Y723C)
8D	ANOVA	14		0.8236		0.539	0.601 (L354P) 0.789 (G521V) 0.999 (P673L) 0.445 (Y723C)
9B	ANOVA	19		19.52		< 0.0001	0.0001 (Sap97‐P1P2) 0.0022 (CASK G521V) 0.0001 (CASK P673L) 0.0001 (CASK Y723C)
9C	ANOVA	23		22.61		< 0.0001	0.0001 (Sap97‐P1P2) 0.0001 (Sap97‐PSG) 0.0001 (CASK G521V) 0.0001 (CASK P673L) 0.0001 (CASK Y723C)
9D	ANOVA	23		26.36		< 0.0001	0.0001 (Sap97‐P1P2) 0.0001 (CASK WT) 0.0001 (CASK G521V) 0.0001 (CASK P673L) 0.0001 (CASK Y723C)

## Author Contributions

**Debora Tibbe:** conceptualization, investigation, writing – original draft. **Hans‐Hinrich Hönck:** investigation. **Neha Bhatia:** data curation, investigation. **Tina Truong:** data curation, investigation. **Lydia Proskauer:** investigation, formal analysis. **Xilma Ortiz‐Gonzalez:** investigation, data curation. **Jean Ann Maguire:** investigation, data curation. **ChangHui Pak:** conceptualization, funding acquisition, supervision. **Hans‐Jürgen Kreienkamp:** conceptualization, funding acquisition, supervision, writing – original draft, writing – review and editing.

## Supporting information


**Appendix S1:** jnc70303‐sup‐0001‐AppendixS1.pdf.

## Data Availability

All data needed to evaluate the conclusions in the paper are present in the paper and/or Appendix S1.
