# Supplementary material for: Patient‐Derived Variants Define Constraints for Ligand Binding at the PDZ Domain of CASK
Source: J Neurochem. 2025 Dec 1;169(12):e70303. doi: 10.1111/jnc.70303 (PMC12666738; doi:10.1111/jnc.70303)
Supplement: Supplementary file 1 — Appendix S1: jnc70303‐sup‐0001‐AppendixS1.pdf. [file JNC-169-0-s001.pdf]

Patient-derived variants define constraints for ligand binding at the PDZ domain of CASK

Debora Tibbe[1], Hans-Hinrich Hönck[1], Neha Bhatia[2], Tina Truong[2], Lydia Proskauer[3], Xilma Ortiz-Gonzalez[4,5], Jean Ann Maguire[6], ChangHui Pak[3] and Hans-Jürgen Kreienkamp[1]

Supplemental material

Supplemental Table 1.

**Subject #**	1
Referring centre	Boston Children's Hospital
Gene	*CASK*
Mutation NM_003688.3	c.2014A>G, p.I672V
Exon	21
Origin (maternally inherited; *de novo*)	Maternally inherited
Nationality	Brazilian ancestry
Sex	M
**Pregnancy and** [illegible]	
Pregnancy	uncomplicated, full-term pregnancy and vaginal
Birth at	40
Birth weight (centile, z-score)	3.57 kg (53rd %tile, Z= 0.5)
Birth length (centile, z-score)	n/a
OFC birth (centile, z-score)	n/a
**Last examination**	
Age	17
Weight (centile, z-score)	68.5 kg (63rd %tile, Z= 0.3)
Height (centile, z-score)	166 cm (10th %ile, Z= -1.2)
OFC (centile, z-score)	54.1 cm (12th %tile, Z= -1.2)

**Development**	
DD/ID	DD and ID, has an IEP
Motor development	Yes, started walking at 18 months old
Speech impairment	Yes, first understand able words at 3 years old
**Neurological features**	
Muscular hypotonia and/or hypertonia	Hypotonia
Seizures	No
Seizure onset	n/a
Seizure type	n/a
EEG	n/a
Response to treatment	n/a
MRI or CT scan (provide age and abnormalities)	Normal Brain MRI/MRA w/o contrast at age 14 years old
**Other findings**	
Hearing	Normal hearing evaluation at 16 years old
Eye findings	Astigmatism , hyperopia wears glasses
Feeding	
Craniofacial dysmorphism	Arched eyebrows, otherwise nondysmor phic

Additional findings	Autism, ADHD, Slipped capital femoral epiphysis of the left hip with subsequent deformity of the proximal femur and limb length discrepancy s/p repair, Scoliosis, likely pathogenic variant in COL1A2

Supplemental Figure 1.

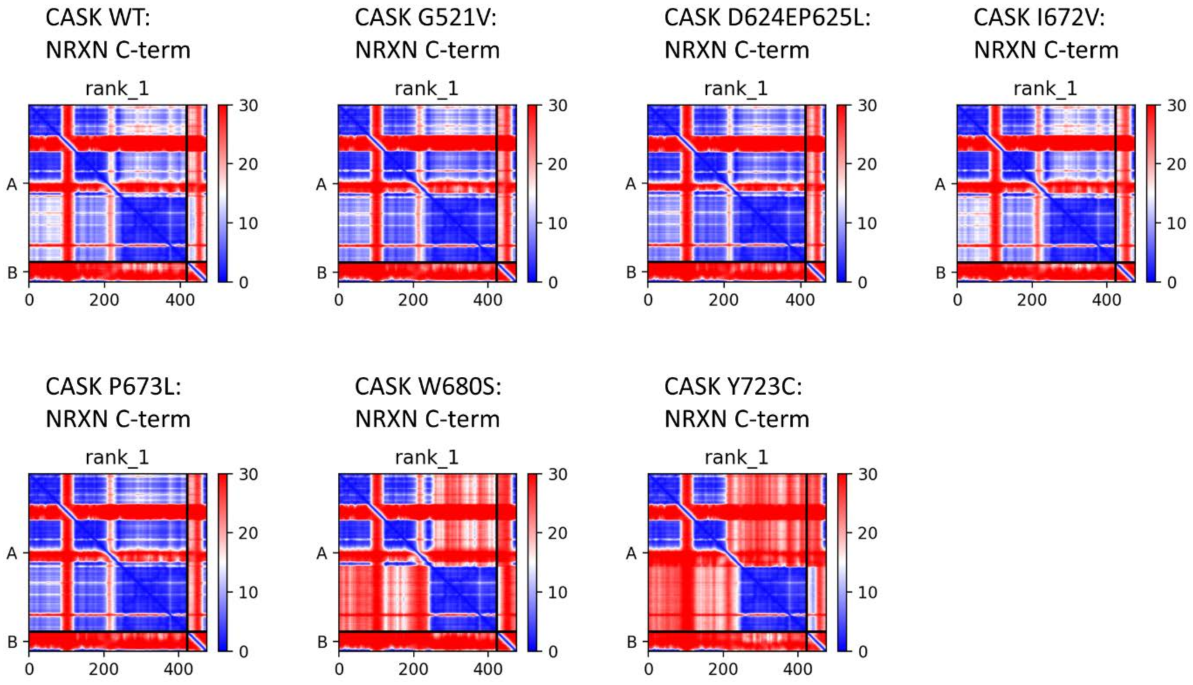

Supplemental Figure 1: AlphaFold predictions for complexes between CASK variants and the NRXN1 intracellular C-terminal domain.

Representative predicted aligned error (PAE) plots for the highest ranked predicted models of the CASK superdomain in complex with the C-terminus of NRXN1. Protein sequences were submitted to the Colabfold software to predict complex formation. Alphafold 2.2 confidence measures of the highest ranked complex predictions are represented in PAE plots. Residue numbers are displayed on the X-axis and PAE values suggesting the confidence that two residues are aligned are represented by the colored scale, with high confidence alignments displaying a lower PAE value. Note that the W680S and Y723C variants elicit low confidence in areas where the C-terminal GK domain contacts the SH3 and PDZ domains of CASK (lower left and upper right quadrants). Protein sequences used for modeling: CASK: O14936, residues residues 496-912; NRXN1: P58400, residues 420-472.

Supplemental material: full, original, uncropped Western Blots

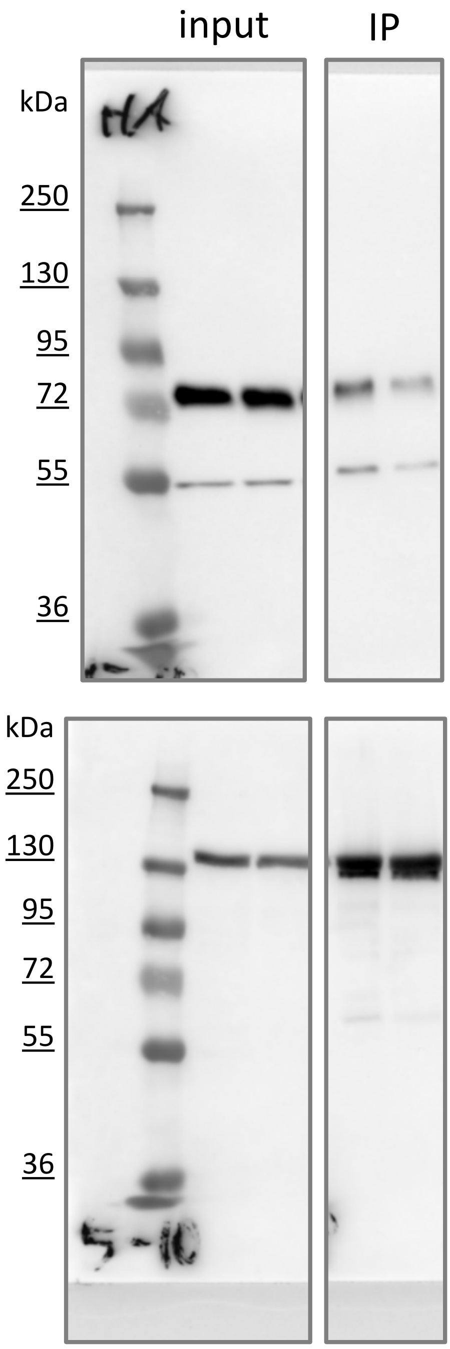

Figure 2A
antiHA (Nrxn1)

Figure 2A
antiRFP (CASK)

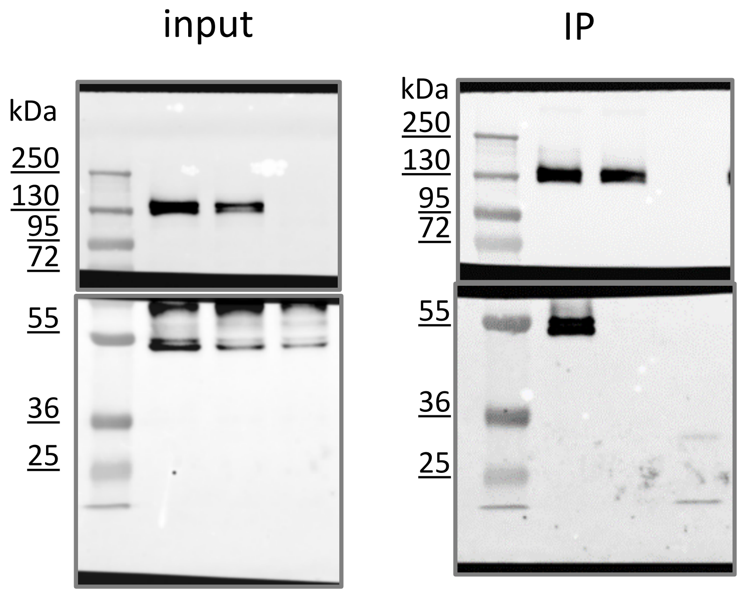

Figure 2C
antiRFP (CASK)

Figure 2C
antiHA (Nrxn1)

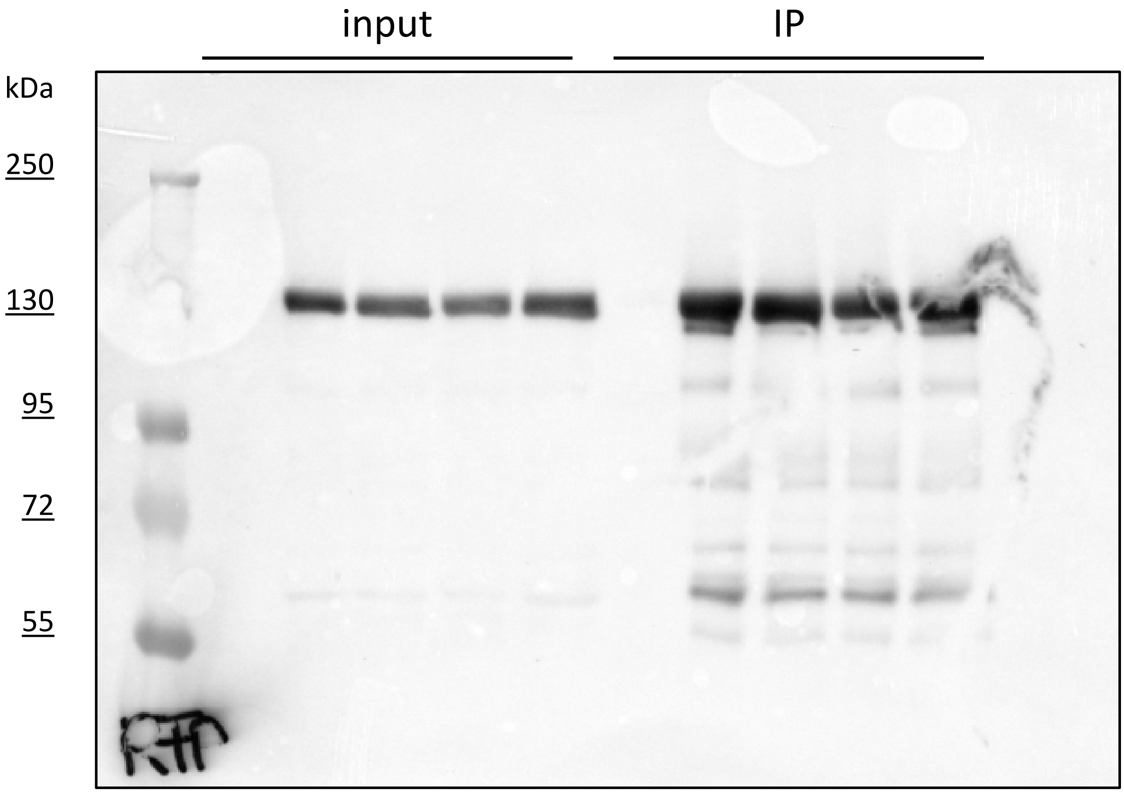

Figure 3a; anti-CASK

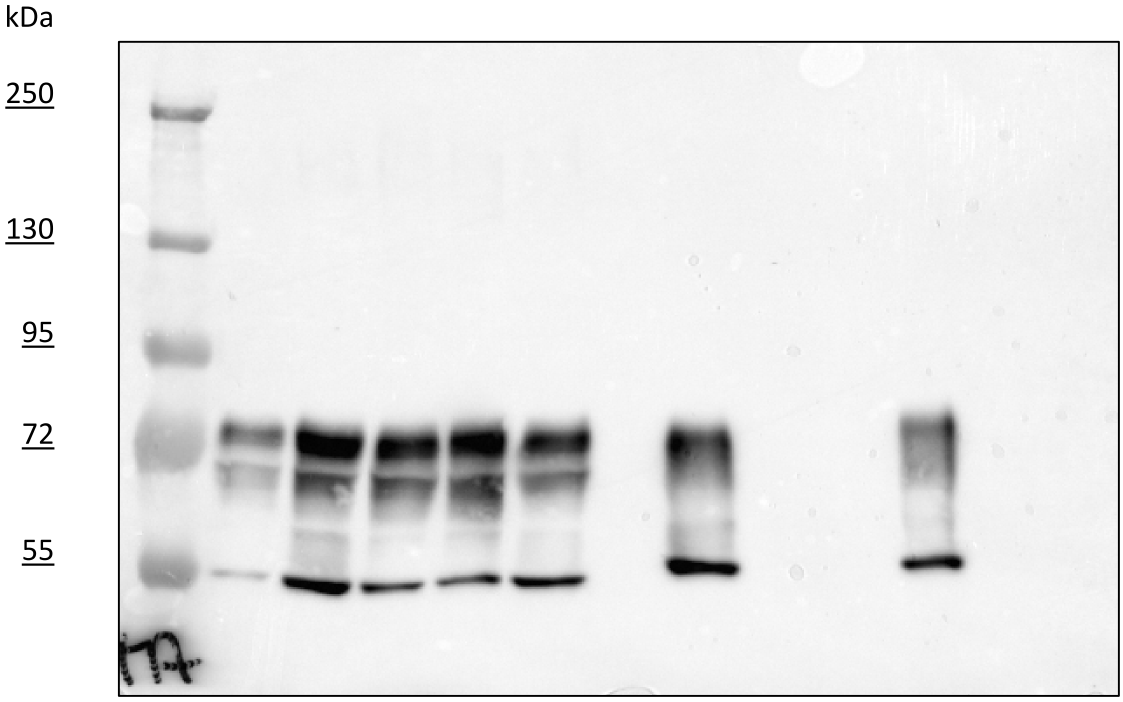

Figure 3a; anti-HA (Nrxn1)

Figure 5a

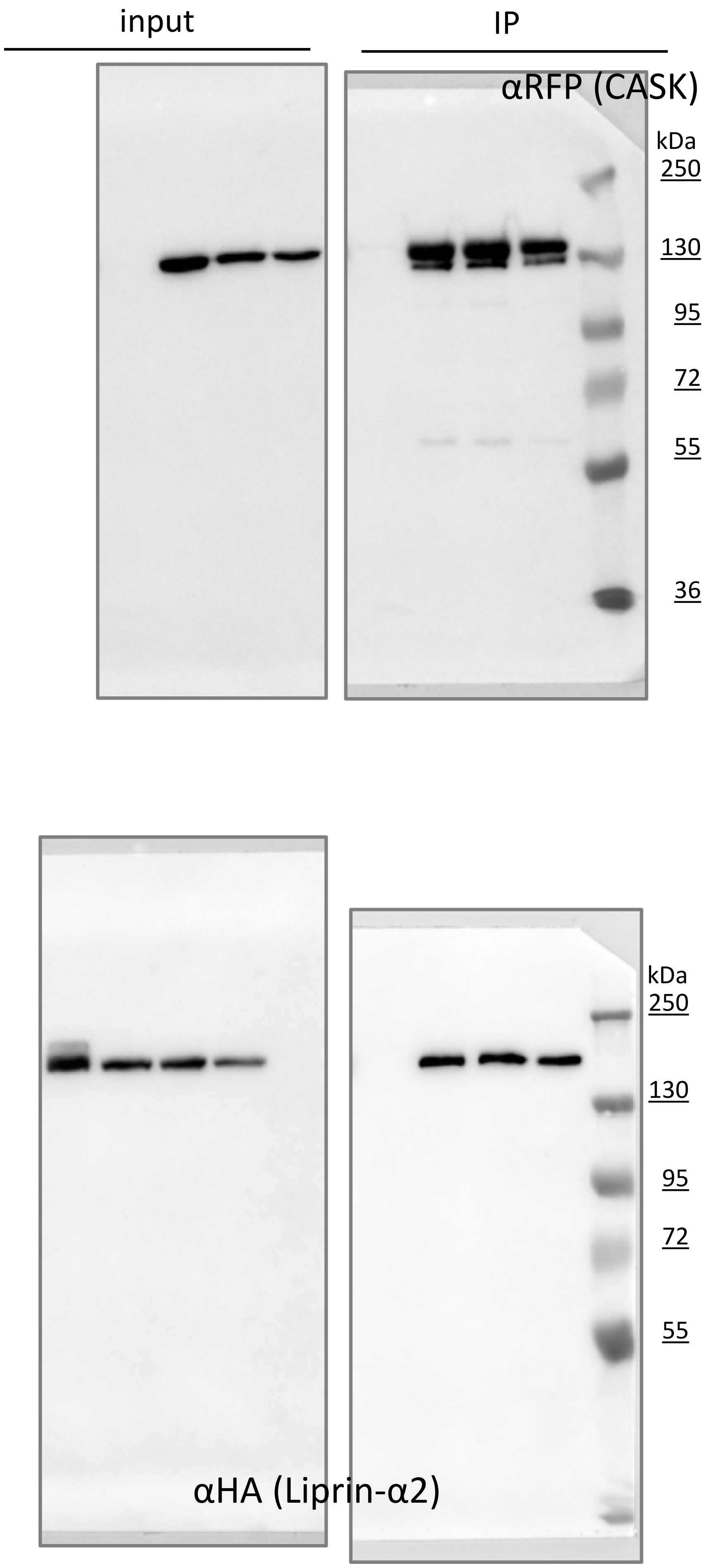
input
IP
αRFP (CASK)
kDa
250
130
95
72
55
36
kDa
250
130
95
72
55
αHA (Liprin-α2)

Figure 5c, left

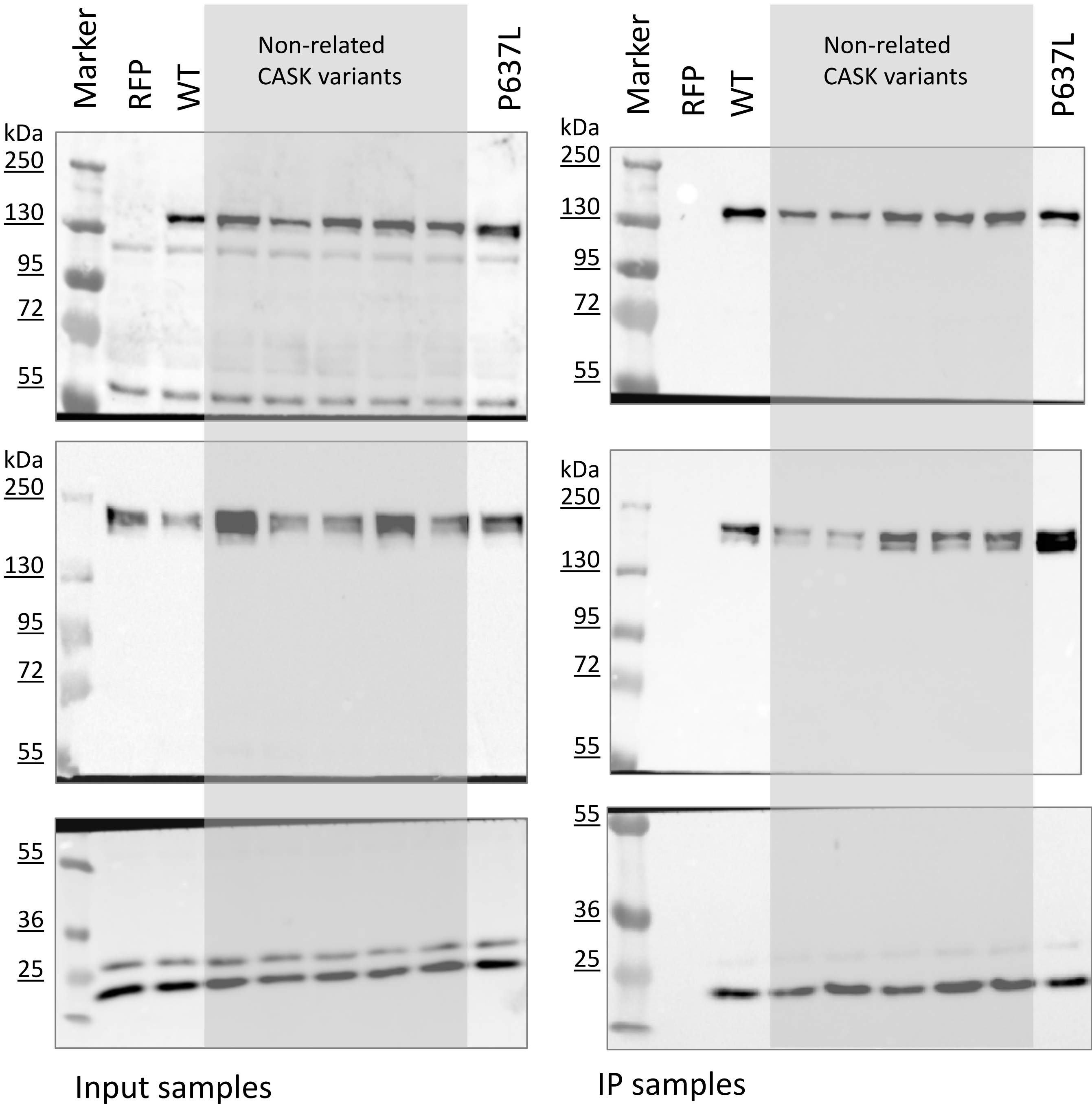

Figure 5c, right

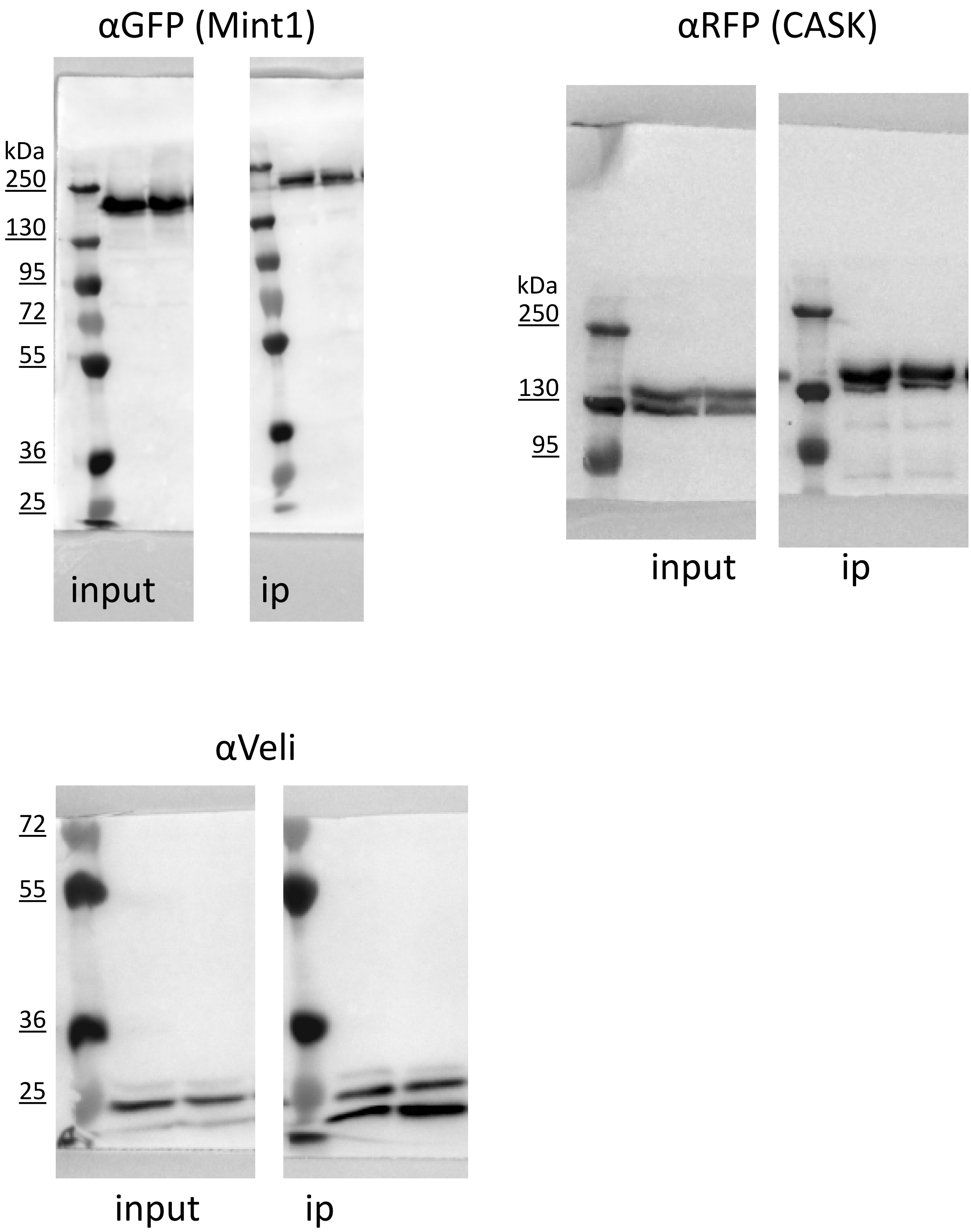

Figure 7A

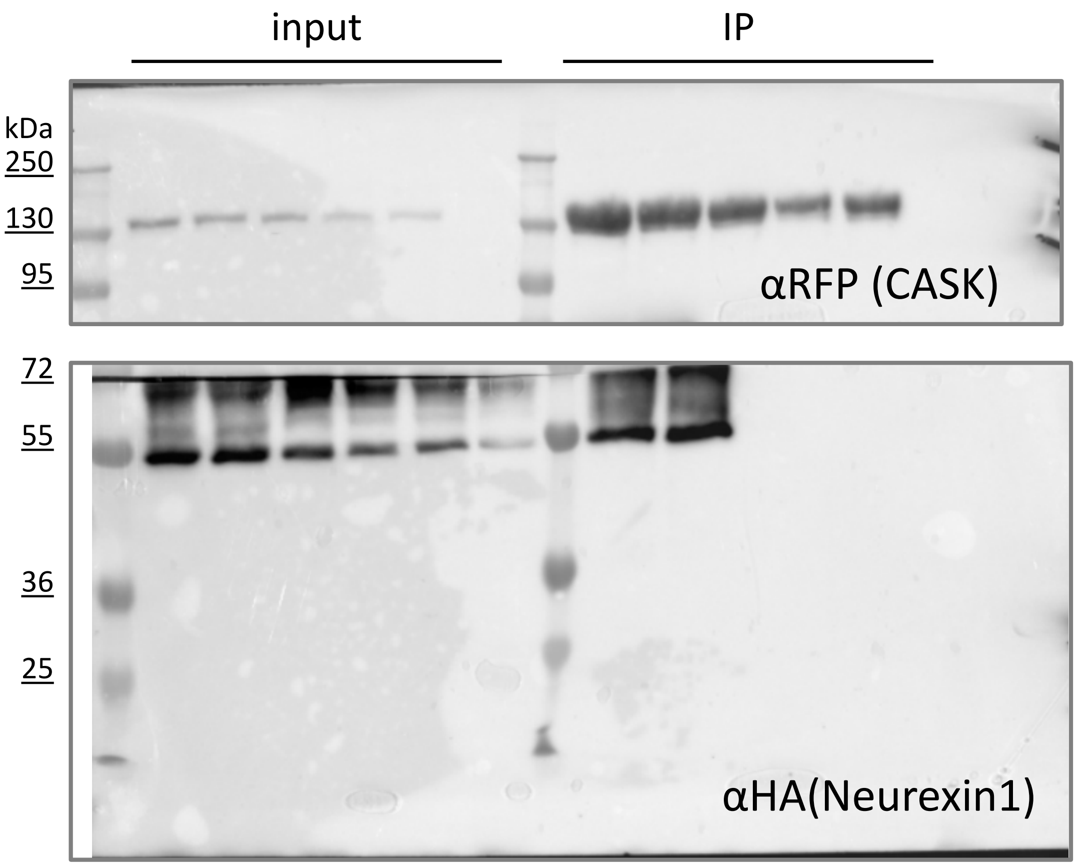

Figure 7B

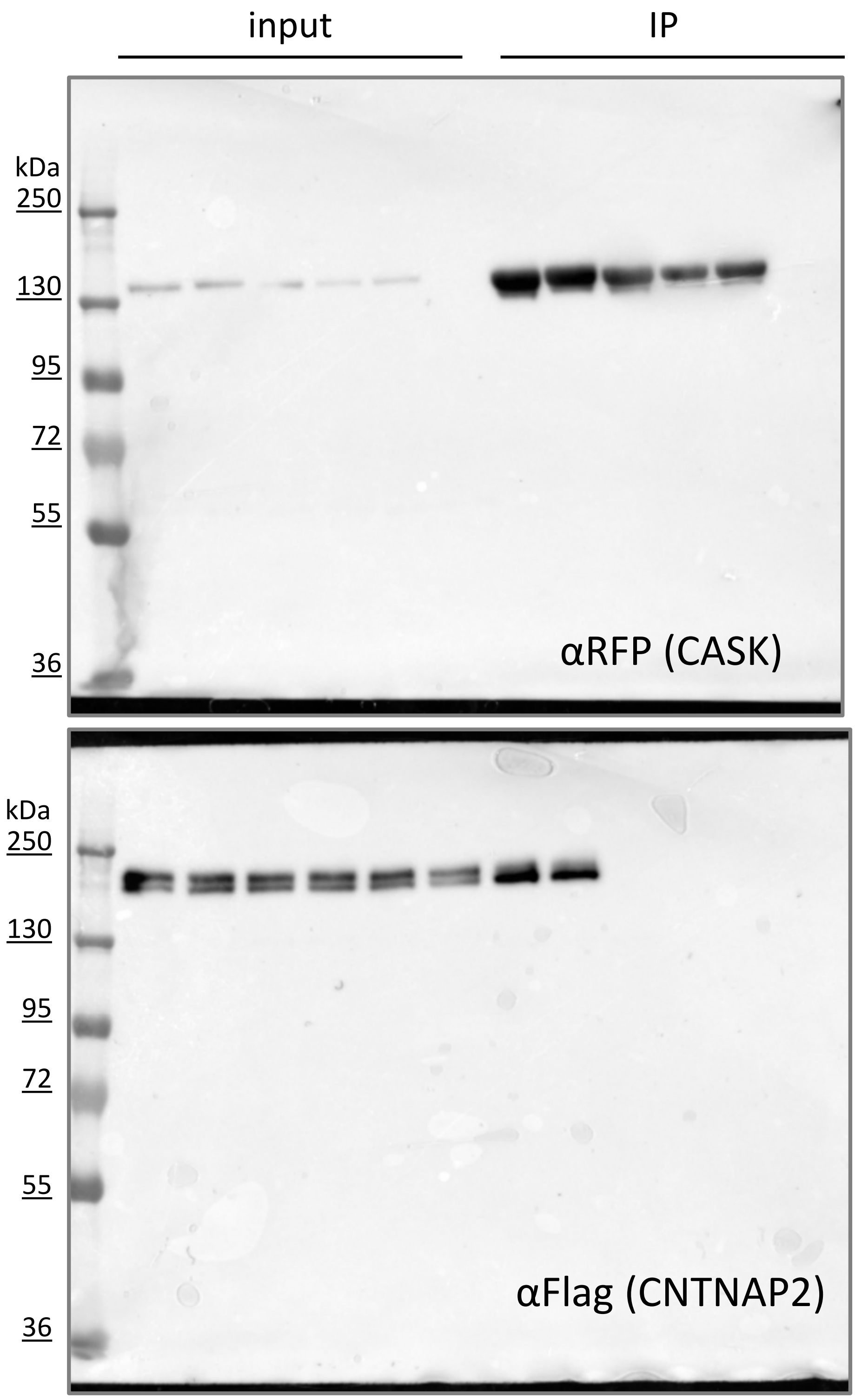
input
IP
kDa
250
130
95
72
55
36
αRFP (CASK)
kDa
250
130
95
72
55
36
αFlag (CNTNAP2)

Figure 8A

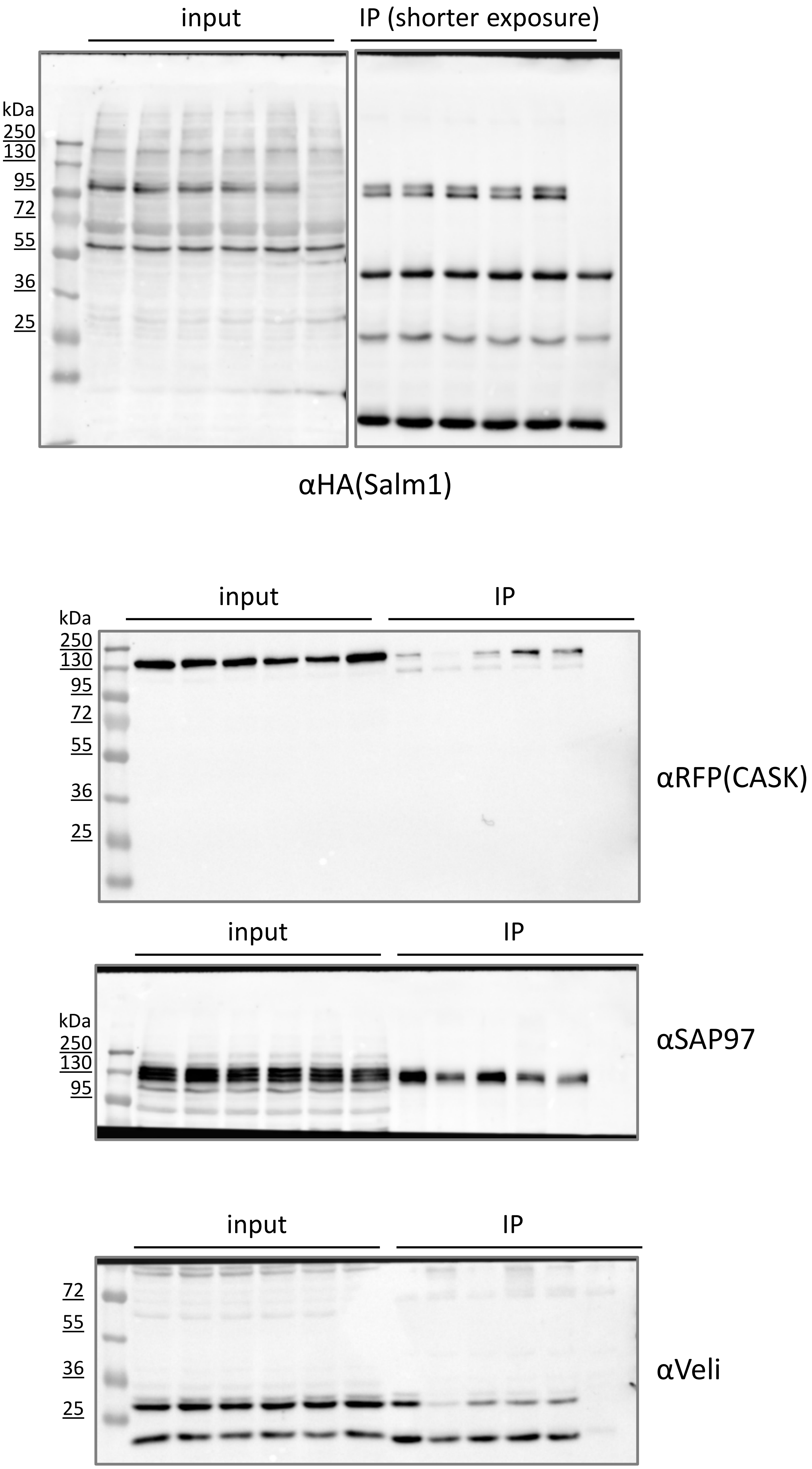
input
IP (shorter exposure)
kDa
250
130
95
72
55
36
25
αHA(Salm1)
input
IP
kDa
250
130
95
72
55
36
25
αRFP(CASK)
input
IP
kDa
250
130
95
αSAP97
input
IP
72
55
36
25
αVeli

Figure 9A

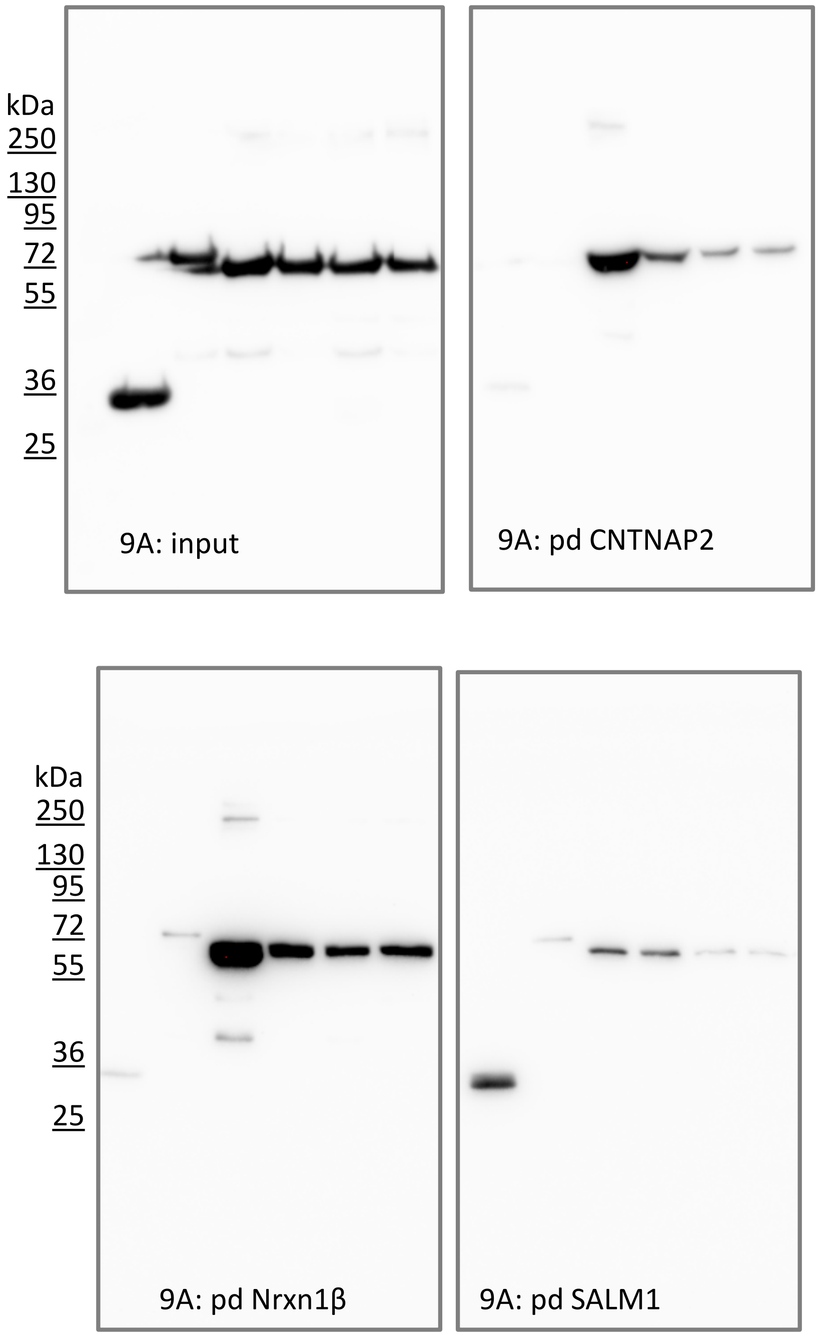
kDa
250
130
95
72
55
36
25
9A: input
9A: pd CNTNAP2
kDa
250
130
95
72
55
36
25
9A: pd Nrxn1β
9A: pd SALM1
